# Supplementary material for: Feasibility study of a multicentre cluster randomised control trial to investigate the clinical and cost-effectiveness of a structured diagnostic pathway in primary care for chronic breathlessness: protocol paper
Source: BMJ Open. 2021 Nov 23;11(11):e057362. doi: 10.1136/bmjopen-2021-057362 (PMC8611440; doi:10.1136/bmjopen-2021-057362)
Supplement: Supplementary data [file bmjopen-2021-057362supp001.pdf]

FUNDED BY NIHR | National Institute for Health Research — UNIVERSITY OF LEICESTER — NHS University Hospitals of Leicester NHS Trust — Breathlessness Diagnostic Pathway

CONSENT FORM

Breathlessness Study – Diagnostic Pathway

**Site: Glenfield Hospital**	**Participant ID No:**
**Sponsor: University of Leicester**	**Name of researcher:**

Please initial in the box

1. I confirm that I have read and understand the Participant Information Sheet **version no. ___dated** ____for the above study, and have had the opportunity to ask questions. ☐
2. I understand that my participation is voluntary and that I am free to withdraw at any time without giving a reason, and without my medical care or legal rights being affected. I understand that any data collected up until my withdrawal will still be used in the research study. ☐
3. I agree to undergo the tests and investigations described in the Participant Information Sheet. The nature of the tests, investigations and any possible risks have been explained. ☐
4. I understand that relevant sections of my medical records and data collected during the study may be looked at by individuals from the study team, Leicester Clinical Trials Unit, the Sponsor, regulatory authorities or the NHS Trust, where it is relevant to my taking part in this research. I give permission for these individuals to have access to my records. ☐
5. I give permission to the study team to retrieve data about the healthcare services I have utilised over the 12 months of the study. ☐
6. I understand that the information held and maintained by the Health and Social Care Information Centre (NHS Digital) and other central UK NHS bodies may be accessed by research team to obtain information about my health status at one and five years. I give permission to the study team to access the data by linking it to my identifiable information. ☐
7. I agree to my GP being informed of my participation in the study. ☐
8. I understand that my personal details and study data will be stored on secure University of Leicester and University Hospitals of Leicester systems and paper copies will be kept in a secure office environment at Glenfield Hospital. ☐
9. I agree that the information collected about me will be used to support other research in the future, and may be shared anonymously with other researchers. ☐
10. I agree that some of my anonymised data will be shared with providers of the questionnaire license holders for the purpose of improving how the questionnaires are used. ☐
11. I agree to take part in an interview to find out about my experiences of breathlessness and healthcare. I understand interviews will be recorded and transcribed. ☐
12. I agree to take part in the above study. ☐

Name of participant (printed)	Date	Signature
Name of person taking consent (printed)	Date	Signature

When completed: 1 for participant; 1 (original) for ISF; 1 to GP

Breathlessness Diagnostic Pathway_Consent form_v1.1_2020-02-05 IRAS ID: 261499

Breathlessness Diagnostic Pathway

Study aims

- To conduct nested qualitative interviews with patients and clinicians to understand how **acceptable the trial design** is including using the pathway, patient research visits and use of outcome measures.
- To conduct nested interviews to **understand more about patient experiences of breathlessness, the process of diagnosis and how it affects their quality of life.**
- To also understand **from clinicians** more about 'usual care' and current practices and attitudes in primary care towards breathlessness as a symptom.

Interview Schedule: Understanding patient experiences in taking part in the Breathlessness Diagnostic Pathway study

Introduce self: name and role

Withdrawal

If anything we speak about today makes you feel uncomfortable you are free to not answer a particular question, request for the recorder to be switched off to resume the interview after a short break, or you can ask to terminate the interview all together at any point.

Before we start the interview I would like to collect some information about yourself:

Background information from participant: To be recorded on CRF

- **(Covid Y/N, Covid symptoms Y/N)** -maybe leave till later unless they volunteer but ensure it is recorded by end of the interview?

Thank you. We will now proceed to the interview:

Seek consent to continue and to audio-record the interview.

Let them know that no personal identifiable data will be recorded and a participant number will be allocated to them. There are no right or wrong answers here; we are just trying to understand what things are like for you.

Opening (to understand what the patient knows about the trial and has done so far in terms of visits).

- **Can you talk me through why you're here?/ What you think the study is about?/How you started on the study?**
  - What have you done so far? How has that been?

1: Experiences of breathlessness

- Can you tell me about your breathlessness?

  [**prompt** – I really want to understand what its like for you…. Can you tell me a bit more about that…]

2: COVID-19

- **Can you tell me about your normal day at the moment?**
- **How are you managing**? (with the current situation)

  (Going out? Social shielding? Contact with others? Anxiety?)

  - Can you describe what your health is like at the moment?

  (Symptoms of covid?)

  - How are you keeping in contact with people/if anyone?
  - Does the patient mention their breathlessness with regards to government guidelines here? If not, prompt?
  - If not reported prompts: coping, exercise, social life, worrying, depressed, isolated, existential crisis/future worries, and if seeking help – how?

- Can you tell me about any changes to your health care in the current situation (COVID-19)?

  (e.g. prescriptions, routine appointments)

Possible prompt (How is the current pandemic affecting you?

3: Experiences of breathlessness

- Thinking back, leading up to you going to the GP, can you talk me through how breathlessness was affecting you? [what led you to go to the GP?]
  - Can you tell me about when you first noticed you were becoming more breathless?
  - What made you visit your GP?
    - (Reasons, worries, what did they think might be causing it, ? prompted by family, ? stopped being able to manage certain things/jobs)
    - (Did they go for breathlessness or was it something else and GP picked up the breathlessness?)

Doe G, *et al. BMJ Open* 2021; 11:e057362. doi: 10.1136/bmjopen-2021-057362

- What were you expecting might happen?
- Can you tell me what think is causing your breathlessness?
  - What do you understand about that?

4: Healthcare experiences

- **Thinking about your breathlessness**, can you talk me through what's happened so far? Medical pathway / who you've been to see to, tests done, diagnosis ?
  - What do you understand about the diagnosis**?**

5: Lifestyle

- Does anything help you with your breathlessness? (are they actively coping? If so where have they found out how to do this, where have they looked for information)
  - Where did you come across this? information source /worked it out themselves?
  - Has it changed since seeking help?
  - How does it affect your everyday life?

6: Experience of taking part in the study

- In relation to this research study, what has been your experience of taking part?
  - What were the positive things?
  - What has been difficult about taking part?
  - Is there anything you think could be changed

Is there anything that we haven't covered in the interview that you think we should know or think about?

<u>Interview Schedule:</u> Understanding GP experiences in taking part in the Breathlessness Diagnostic Pathway study

Introduce self: name and role

Let them know that no personal identifiable data will be recorded and a participant number will be allocated to them.

Withdrawal

If anything we speak about today makes you feel uncomfortable you are free to not answer a particular question, request for the recorder to be switched off to resume the interview after a short break, or you can ask to terminate the interview all together at any point.

Before we start the interview I would like to collect some information about yourself: (Background information from participant: To be recorded)

Thank you. We will now proceed to the interview: *Seek consent to continue and to audio-record the interview.*

<u>General/context</u>

- Can you tell me a bit about how you manage breathlessness?
  - What difficulties/problems/find hard about breathlessness?
    OR
  - Is there anything you find difficult about breathlessness?
- Tell me about the last person you managed with breathlessness OR Can you think of a challenging experience with a patient with breathlessness? OR Can you tell me an instance where arriving at a diagnosis for a patient with chronic breathlessness has been difficult?
  - *Why was it challenging?*
  - *Feelings, worries, support*

(Try to see if they are focusing on disease or on breathlessness management too? Note for interviewer…)

Tell me how it really works in practice (verses ideal world)

What about managing the symptoms for that patient?

Can you tell me what you do if/when you've run all your tests and you don't find a particular cause/reason for their breathlessness?

And how do you phrase that information to the patient?

- How do you make decisions about referral?
  - *How do you make a decision that this person's breathlessness merits assessment and further management?*

- Thinking about **assessment**: How do you think your colleagues feel about assessing patients with breathlessness? (Do you think your assessment is different to your colleagues?)
- Are there conditions that you or colleagues worry about missing or are harder to pick up?
  - Why is that?

- Thinking about breathlessness **management**: How do you go about managing patients with breathlessness? (Do you colleagues work in s similar/different way?)

Breathlessness Diagnostic Pathway_GP Interview Schedule_v5.2.docx 2021-07-06 IRAS ID: 261499

- Who else might help with the patient management (in the practice, in the area, onward referral, PR)?

  (Possible prompt: what is it that makes breathlessness different as a symptom to say back pain for example in terms of how it is assessed?)

- Thinking about patients living with chronic breathlessness: in your experience are patients coming to seek help because it has got worse (urgently seeking help) or coming in with another problem? What do you think………

Thinking about the study specifically and the diagnostic pathways for breathlessness

- What are the barriers to doing investigations or making a diagnosis?
- What would be a red flag that would make certain investigations more of priority or increase the urgency of getting them done?

What do you think an effective pathway for breathlessness should look like?

- Spirometry
- Diagnostic Hubs
- Referral
- Coding for breathlessness
- *Tests eg. CXR, blood tests – are they helpful? Where does that get you?*

  What would help or influence your clinical practice with patients with breathlessness?

Covid-19 (this might get picked up earlier in discussion)

- How are you managing with routine care within the practice at present? (Workload, structural changes/processes)
- How do you think patients are managing?
- How are you finding things are going with your patients with chronic breathlessness under the present conditions?
- How do think assessment or diagnosis is affected by COVID?

Any changes in everyday practice

In relation to this research project – can you tell me about your experience of taking part in the study so far?

Can you talk me through how the pop up worked for you?

- *Intervention: Can you tell me how being in the trial has altered your practice?*
- *Usual care: Can you tell me if being in the trial has had any effect on your practice?*

About the study

- In relation to this research study, what has been your experience of taking part?
  - Is there anything you think could be changed?

Is there anything that we haven't covered in the interview that you think we should know or think about?
