# Supplementary material for: Feasibility study of a multicentre cluster randomised control trial to investigate the clinical and cost-effectiveness of a structured diagnostic pathway in primary care for chronic breathlessness: protocol paper
Source: BMJ Open. 2021 Nov 23;11(11):e057362. doi: 10.1136/bmjopen-2021-057362 (PMC8611440; doi:10.1136/bmjopen-2021-057362)
Supplement: Author's manuscript [file bmjopen-2021-057362.draft_revisions.pdf]

BMJ Open

BMJ Open is committed to open peer review. As part of this commitment we make the peer review history of every article we publish publicly available.

When an article is published we post the peer reviewers' comments and the authors' responses online. We also post the versions of the paper that were used during peer review. These are the versions that the peer review comments apply to.

The versions of the paper that follow are the versions that were submitted during the peer review process. They are not the versions of record or the final published versions. They should not be cited or distributed as the published version of this manuscript.

BMJ Open is an open access journal and the full, final, typeset and author-corrected version of record of the manuscript is available on our site with no access controls, subscription charges or pay-per-view fees (http://bmjopen.bmj.com).

If you have any questions on BMJ Open's open peer review process please email info.bmjopen@bmj.com

BMJ Open

A protocol for a feasibility study of a multi-centre cluster randomised control trial to investigate whether a structured diagnostic pathway in primary care is clinically and cost effective for adults presenting with chronic breathlessness.

Journal:	*BMJ Open*
Manuscript ID	bmjopen-2021-057362
Article Type:	Protocol
Date Submitted by the Author:	14-Sep-2021
Complete List of Authors:	Doe, Gillian; University of Leicester, Respiratory Sciences<br>Clanchy, Jill; University of Leicester<br>Wathall, Simon; Keele University, Institute of Primary Care & Health Sciences<br>Chantrell, Stacey; University Hospitals of Leicester NHS Trust<br>Edwards, Sarah; University Hospitals of Leicester NHS Trust<br>Baxter, Noel; International Primary Care Respiratory Group<br>Jackson, Darren; Barwell & Hollycroft Medical Centres<br>Armstrong, Natalie ; University of Leicester, Health Sciences<br>Steiner, Michael; Institute for Lung Health, NIHR Leicester Biomedical Research Centre – Respiratory, University of Leicester, Glenfield Hospital,<br>Evans , Rachael A; University of Leicester, Department of Respiratory Science; University Hospitals of Leicester NHS Trust
Keywords:	PRIMARY CARE, RESPIRATORY MEDICINE (see Thoracic Medicine), QUALITATIVE RESEARCH, Quality in health care < HEALTH SERVICES ADMINISTRATION & MANAGEMENT

SCHOLARONE™
Manuscripts

I, the Submitting Author has the right to grant and does grant on behalf of all authors of the Work (as defined in the below author licence), an exclusive licence and/or a non-exclusive licence for contributions from authors who are: i) UK Crown employees; ii) where BMJ has agreed a CC-BY licence shall apply, and/or iii) in accordance with the terms applicable for US Federal Government officers or employees acting as part of their official duties; on a worldwide, perpetual, irrevocable, royalty-free basis to BMJ Publishing Group Ltd ("BMJ") its licensees and where the relevant Journal is co-owned by BMJ to the co-owners of the Journal, to publish the Work in this journal and any other BMJ products and to exploit all rights, as set out in our licence.

The Submitting Author accepts and understands that any supply made under these terms is made by BMJ to the Submitting Author unless you are acting as an employee on behalf of your employer or a postgraduate student of an affiliated institution which is paying any applicable article publishing charge ("APC") for Open Access articles. Where the Submitting Author wishes to make the Work available on an Open Access basis (and intends to pay the relevant APC), the terms of reuse of such Open Access shall be governed by a Creative Commons licence – details of these licences and which Creative Commons licence will apply to this Work are set out in our licence referred to above.

Other than as permitted in any relevant BMJ Author's Self Archiving Policies, I confirm this Work has not been accepted for publication elsewhere, is not being considered for publication elsewhere and does not duplicate material already published. I confirm all authors consent to publication of this Work and authorise the granting of this licence.

For peer review only

Word Count = 3853

Title: A protocol for a feasibility study of a multi-centre cluster randomised control trial to investigate whether a structured diagnostic pathway in primary care is clinically and cost effective for adults presenting with chronic breathlessness.

(Breathe DEEP: Breathlessness – DiagnosE Early in Primary Care.)

Authors:

Ms Gillian Doe[1], Ms Jill Clanchy[2], Mr Simon Wathall[3], Ms Stacey Chantrell[4], Ms Sarah Edwards[4], Dr Noel Baxter[5], Dr Darren Jackson[6], Prof Natalie Armstrong[7], Prof Michael Steiner[1,4] and *Dr Rachael A Evans[1,4]

Institutions:

1. Department of Respiratory Science, University of Leicester, Leicester, UK.

2. Clinical Trials Unit, University of Leicester, Leicester, UK.

3. Clinical Trials Unit, Keele University, UK.

4. NIHR Biomedical Research Centre – Respiratory theme, University Hospitals of Leicester NHS Trust, Leicester, UK.

5. International Primary Care Respiratory Group, UK.

6. Barwell & Hollycroft Medical Centres, Chair Hinckley & Bosworth Medical Alliance.

7. Department of Health Sciences, University of Leicester, Leicester, UK.

For peer review only

For peer review only - http://bmjopen.bmj.com/site/about/guidelines.xhtml

Author for correspondence: *Dr Rachael Evans, Leicester NIHR Biomedical Research Centre – Respiratory, Glenfield Hospital, Leicester, LE3 9QP (Email: re66@leicester.ac.uk). ORCID ID: https://orcid.org/0000-0002-1667-868X

Abstract

Introduction: Chronic breathlessness is a common and debilitating symptom, associated with high healthcare use and reduced quality of life. Challenges and delays in diagnosis for people with chronic breathlessness frequently occur, leading to delayed access to therapies. The overarching hypothesis is a symptom-based approach to diagnosis in primary care would lead to earlier diagnosis, and therefore earlier treatment and improved longer term outcomes including health-related quality of life.

This study aims to establish the feasibility of a multicentre cluster randomised controlled trial to assess the clinical and cost effectiveness of a structured diagnostic pathway for breathlessness in primary care.

Methods and Analysis: Ten General Practitioner (GP) practices across Leicester and Leicestershire will be cluster randomised to either a structured diagnostic pathway (intervention) or usual care. The structured diagnostic pathway includes a panel of investigations within one month. Usual care will proceed with patient care as per normal practice. Eligibility criteria include patients presenting with chronic breathlessness for the first time, who are over 40 years old and without a pre-existing diagnosis for their symptoms. An electronic template triggered at the point of consultation with the GP will aid opportunistic recruitment in primary care.

For peer review only

For peer review only - http://bmjopen.bmj.com/site/about/guidelines.xhtml

The primary outcome for this feasibility study is recruitment rate. Secondary outcome measures, including time to diagnosis, will be collected to help inform outcomes for the future trial and to assess the impact of an earlier diagnosis. These will include symptoms, health-related quality of life, exercise capacity, measures of frailty, physical activity and healthcare utilisation.

The study will include nested qualitative interviews with patients and healthcare staff to understand the feasibility outcomes, explore what is 'usual care' and the study experience.

Ethics and Dissemination: The Research Ethics Committee Nottingham 1 has provided ethical approval for this research study (REC Reference: 19/EM/0201).

Trial registration: ISRCTN14483247

Strengths and Limitations:

- A mixed methods approach will be used to both understand how breathlessness is perceived and diagnosed in primary care, and further interpret the findings of the feasibility study.
- The structured diagnostic pathway uses existing investigations available in primary care.
- The trial is embedded within clinical care and utilises opportunistic recruitment when patients present with breathlessness in primary care.
- The study design is a cluster randomised trial.
- This trial will be conducted in a single region in the UK.

For peer review only - http://bmjopen.bmj.com/site/about/guidelines.xhtml

Introduction

Breathlessness is associated with high healthcare use, accounting for 5% of presentations to the emergency department (1, 2), approximately 4% of GP consultations (3) and reported by patients in 12% of medical admissions (4). Breathlessness is reported by around 9-11% (5, 6) of the general population, varying with severity, socioeconomic status (6, 7) and increasing with age to 25% in people over seventy years old (8, 9). Functional impairment from breathlessness, measured using the Medical Research Council (MRC) dyspnoea scale, is associated with reduced survival regardless of underlying diagnosis (10).

Two-thirds of breathlessness is caused by cardiorespiratory conditions (11). Clinical data shows that for patients over the age of forty the most common causes of breathlessness are Chronic Obstructive Pulmonary Disease (COPD), heart failure (HF), obesity, anaemia and anxiety (12, 13). These conditions can be potentially diagnosed or excluded using investigations that can be performed in community settings (12). All of these investigations are recommended in the National Institute for Health and Care Excellence (NICE) clinical knowledge summary (14) but there is no specific guidance on the timeframe within which they should be performed.

Primary care data have highlighted many missed opportunities over many years to diagnose conditions associated with breathlessness, such as COPD and HF (15, 16), with many patients being diagnosed only when the disease is severe or requiring hospitalisation (15, 16). These data indicate significant challenges in the deployment of simple diagnostics in the primary care setting. There are also well-documented misdiagnoses for COPD, asthma and interstitial Lung Disease across healthcare settings (15, 17-21).

For peer review only - http://bmjopen.bmj.com/site/about/guidelines.xhtml

Our overarching hypothesis is that a symptom-based approach for diagnosis in primary care for patients with chronic breathlessness will lead to earlier diagnosis, earlier treatment and improved outcomes such as health-related quality of life. However, it is also important to consider the implications of over investigating and 'over-diagnosis' in patients and find the balance between clinical and cost effectiveness for a diagnostic pathway (22). A large and potentially expensive multi-centre cluster randomised controlled trial would be necessary to understand the clinical and cost effectiveness of a structured diagnostic pathway for chronic breathlessness. The scope of this study is to assess the feasibility of such a trial and help inform the design.

For this feasibility study the specific aims are:

1. To assess feasibility by recruitment and retention rate of patients in the trial to enable calculation of the number of GP practices, cluster sizes and duration of the ultimate RCT (Table 1).

2. To better understand 'usual care' through prospective observation and qualitative analysis, and to understand any influence of the trial design on usual care.

3. To determine the proposed primary outcome measure for the future trial and to increase understanding of what is an important and realistic difference whilst exploring potential of other outcome measures (Table 2).

4. Identify sources of data and how best to collect these in order to plan the economic evaluation that would accompany a full trial.

For peer review only - http://bmjopen.bmj.com/site/about/guidelines.xhtml

Table 1. Feasibility measures

Feasibility measures
Number of patients recruited per week per GP practice population size
Number of participating GP practices verses the number approached
Time for GPs to screen for eligibility
Number of eligible patients who agree to be approached by the research team verses total number of eligible patients
Number and timing of investigations in the diagnostic pathway completed
Acceptability of the research visit to the participants
Data collected from Interviews regarding participant experience of the trial

Table 2. Secondary outcome measures

Secondary Outcome measures	Measurement Tool
Proportion of diagnoses in Usual care and Intervention within one year of presentation	Review of healthcare records for all participants
Time to diagnosis	Review of healthcare records for symptom presentation and diagnosis date
Health-related quality of life	Chronic Heart Questionnaire (CHQ)<br>Euroqol 5 Dimension 5 Level (EQ5D-5L)
Breathlessness	Dyspnoea -12<br>Multidimensional Dyspnoea Profile (MDP)<br>Medical Research Council (MRC) dyspnoea scale<br>Baseline Dyspnoea Index (BDI) and Transition Dyspnoea Index (TDI)
Physical Activity	Activity monitors (GENEActiv and ActiGraph devices) to measure daily step count, sedentary time, moderate and vigorous activity
Exercise capacity	Incremental Shuttle Walk Test (ISWT)
Frailty	Short Performance Physical Battery (SPPB), Fried's frailty score, Rockwood frailty score, handgrip and quadriceps strength.

For peer review only - http://bmjopen.bmj.com/site/about/guidelines.xhtml

Methods and Analysis

This is a mixed methods study designed using the Medical Research Council Guidelines on developing complex interventions (23).

Trial Design and Registration

This is a one-year feasibility cluster randomised controlled trial recruiting from primary care. Ten GP practices from East and West Leicestershire and Leicester City clinical commissioning groups (CCG) will be cluster randomised to a structured diagnostic pathway or usual care. The intervention practices will follow a structured diagnostic pathway to include early investigations. Usual care will continue without any intervention.

The University of Leicester will act as study sponsor and the trial has been registered on the ISRCTN website (ISRCTN14483247).

Patient and Public Involvement

Prior to the trial design an engagement event was held with clinicians and patients from relevant services, including GPs, community and hospital clinicians with cardiorespiratory background, and patients with experience of chronic breathlessness to discuss the optimal breathlessness pathway using Listening into Action (24). The structured pathway to be used in the trial was the output from this engagement, the NICE guidance and the Breathlessness IMPRESS Tips for Clinicians guidance (12, 14).

The NIHR Biomedical Research Centre Patient and Public Involvement (PPI) groups were also consulted about the study design including the duration of the research visits, patient facing information and questionnaire packs. They provided feedback on the type and ordering of the

For peer review only

For peer review only - http://bmjopen.bmj.com/site/about/guidelines.xhtml

questionnaires and ways to reduce burden to patients. The wording for the electronic template to aid recruitment was developed by members of the PPI group. The trial management and steering groups will have patient members. The study team aim to feed back to all local PPI partners with results from this trial.

Participants

Eligibility Criteria for Patients

Patients will be eligible if they are over 40 years old, experienced breathlessness for over two months, and are within their first two presentations to primary care with symptoms of breathlessness. Exclusion criteria are an existing diagnosis for their current symptom of breathlessness, an estimated prognosis of less than one year, or if the patient requires immediate hospitalisation for their symptoms.

Eligibility Criteria for GP practices

GP practices will be approached to take part in this study if they serve a patient population over ten thousand. Practices that are research active, as identified by the local Clinical Research Network (CRN) research scheme, will be approached. The practices will be visited by the study team to discuss taking part in the study and engage with the practice teams.

The GP practices will be randomised 1:1 and stratified by CCG using Statarand (25), a Stata randomisation module (Boston College Department of Economics).

Recruitment

Patients will be recruited over one year. Patients who meet the above eligibility criteria will be approached in primary care when they present with symptoms of chronic breathlessness. An

For peer review only

electronic template on the patient record, triggered at the point of consultation, will be used to aid opportunistic recruitment (see Figure 1). The template will be triggered by either free text or Read codes relating to breathlessness. Limits have been set on the trigger to avoid it appearing for patients who have an existing diagnosis of COPD or HF. The template summarises the study, prompting the GP to ask if the patient gives consent to be contacted by the study team. The GP will select yes or no as appropriate to the patient agreeing to have their contact details sent to the study team.

The electronic template has been developed in partnership with Keele Clinical Trials Unit who will support implementation onto the electronic patient record system (SystmOne and EMIS) for each practice. This approach has been used successfully in other primary care trials (26). The Leicester City Clinical Commissioning Group (CCG) who take the research lead for Leicester and Leicestershire were consulted regarding the use of the electronic template.

[Figure 1.]

Setting

The GP practices are located in Leicester and Leicestershire, England, UK. The research team is based at Glenfield hospital, University Hospitals of Leicester NHS Trust. Participants will be invited to attend Glenfield hospital for a research visit.

Safety Reporting

Participation is considered to be low risk. It is believed the occurrence of any serious adverse events (SAEs) will be low. Participants will be undertaking some physical tests as part of the research. There may be a small risk of worsening breathlessness, changes in blood pressure and changes in heart rate

For peer review only - http://bmjopen.bmj.com/site/about/guidelines.xhtml

and a very small risk of falls. Trained staff and emergency equipment will be available to deal with any serious events. All adverse events and serious adverse events will be recorded on the adverse event log. Any SAEs related to the physical tests conducted as part of the study will be reported.

Procedure

A weekly search will be performed by practice staff to provide a report of patients who have agreed to be contacted by the study team following discussion with the GP, as described above. The report will be sent via secure encrypted nhs.net email and patients will be contacted to complete telephone screening for eligibility. A script for telephone screening will be available for use to confirm patients' age, previous medical history and to explain the study in more detail. If patients agree to hear more about the study they will be sent a participant information sheet by post and, where willing, a provisional appointment letter. When patients attend their research visit, they will complete written informed consent and their GP will be notified of their involvement in the study. For patients who decline to take part in the study or are ineligible, the reasons will be documented and collated.

Where possible, patients will attend their research visit within one month of seeing the GP with their breathlessness symptoms. Patients will attend a second research visit 12 months after their initial appointment and also be contacted by phone at six months and asked to complete the questionnaire pack, which will be sent in the post. Please see figure 2. for the study schedule.

[Figure 2. Study Schedule]

For peer review only - http://bmjopen.bmj.com/site/about/guidelines.xhtml

Intervention – structured diagnostic pathway

Patients who attend GP surgeries in the intervention group will undergo a set of investigations within one month; body mass index (BMI), spirometry, electrocardiogram (ECG), chest X-ray (CXR), Full blood count (FBC), N-terminal (NT)-pro hormone BNP (NT-proBNP) profile, anxiety and depression screening using the Patient Health Questionnaire – 4 item (PHQ-4) (27) and the General Practice Physical Activity Questionnaire (GPPAQ) (28). The GPs and clinicians will be provided with the pathway document to support a structured history, examination and use of the investigations (Figure 3).

For the intervention practices, the electronic template will also prompt the GP to action these investigations. The pathway will recommend that patients are reviewed, along with their results, within one month and appropriate next steps to be taken regarding patient management.

Usual care will also have the electronic template triggered but will only ask the patient for their consent to pass on contact details to the study team. The GPs and clinicians in usual care will be asked to proceed with investigating the patient and their symptoms as per their usual practice and be directed to the NICE Clinical Knowledge summary for Breathlessness(14) to standardise care.

[Figure 3. Diagnostic Pathway]

Outcomes

As this is a feasibility trial, a formal sample size/power calculation is not required. Recruitment itself is one of the main measures of feasibility. The feasibility measures are outlined in Table 1 and the secondary outcome measures in Table 2. Recruitment rate will be recorded as a proportion of

For peer review only - http://bmjopen.bmj.com/site/about/guidelines.xhtml

participants consented compared to the number of participants identified as eligible by the GP practices.

Data Collection

Data will be collected in accordance with Sponsor policies and standard operating procedures. Baseline data will be collected at research Visit 1, within one month of the patient consulting their GP for breathlessness. Questionnaires will be completed by post at six months and Visit 2 will occur at twelve months after Visit 1. Healthcare records will be reviewed for all patients at three and 12 months to record the investigations performed and when, diagnosis, and time to diagnosis.

Health care utilisation data will be recorded at 12 months and five years to provide information on hospital admissions, healthcare use, and patient survival. This information will be collected from patient healthcare records and NHS digital.

Anthropometric Measures

Body Mass Index (BMI) will be calculated by measuring the patient's height and weight. Body composition using bioelectrical impedance will provide measurements of lean mass and body fat percentage. Each participant's waist and hip circumference will be measure to the nearest 0.1cm.

Patient reported outcome measures

Health-related quality of life

The Chronic Heart Questionnaire Self-Report (CHQ-SR) is a validated and responsive questionnaire developed for patients with heart disease to assess health-related quality of life (29). It has four domains: dyspnoea, fatigue, mastery and emotional function and a known minimal clinically important difference (MCID) in patients with chronic heart and lung disease (Chronic Respiratory

For peer review only

Questionnaire version). We will be using it in a population with undifferentiated breathlessness as part of feasibility.

The Euroqol Five Dimension Five Level questionnaire (EQ5D-5L) (30) will be used to assess generic health-related quality of life. The EQ5D-5L was chosen as it is a standardised measure of health status independent of disease used to calculate quality of life year (QALY).

Breathlessness

The following questionnaires will be used to assess different aspects of breathlessness and to help select appropriate patient reported outcome measures for the future trial:

1. Dyspnoea-12 is a brief 12 item, self-complete, questionnaire which has been found to reliably measure breathlessness in a variety of diseases [23]. Dyspnoea has both sensory and afferent components and this tool was developed to ensure both aspects could be measured.

2. The Multi-Dimensional Dyspnoea Profile (MDP) is a self-complete questionnaire for breathlessness divided into an immediate perception domain and an emotional response domain (31). This questionnaire shows responsiveness to change in an acute and routine care setting for patients with breathlessness (31).

3. The Baseline Dyspnoea Index and Transition Dyspnoea Index (BDI/TDI) are short interviewer led questionnaires involving open questions about how their breathlessness affects everyday life (32). This is measured over time in respect to what tasks a patient can manage and how much effort is required to complete a task.

4. The MRC Dyspnoea Grade five point scale is patient completed and requires participants to indicate to what extent their breathlessness limits their function by working down the statements which increase in severity regarding functional limitation. (33).

For peer review only - http://bmjopen.bmj.com/site/about/guidelines.xhtml

Anxiety and depression

Participants will also complete the Hospital Anxiety and Depression (HADS) scale which is a simple self-completed questionnaire with 14 questions relating to either anxiety or depression (34)

Activation measure

The Patient Activation Measure (PAM) is a self-completed questionnaire which assesses patients' knowledge, skills and confidence to manage their own health (35).

Physical Activity

Daily physical activity and stationary time will be assessed for seven days using the GT3x ActiGraph device that is worn around the waist (36), and the wrist worn GENEActiv device (37). Sedentary time, daily step count and time spent in moderate and vigorous physical activity will be recorded. The activity monitors will be fitted at visits 1 and 2 and will be programmed to begin collecting data at midnight. Data will then be collected for seven days thereafter. To maximise the use of the data for comparison with other disease datasets we will use both devices as long as patients are willing. If participants can only use one device we will request that this is the wrist worn GENEActiv.

Exercise capacity

This will be assessed using the Incremental Shuttle Walk Test (ISWT) (38). This walking test requires the patient to walk between two cones nine metres apart in time to a set of auditory beeps. Initially, the walking speed is very slow, but each minute the required walking speed progressively increases. The patient will walk for as long as they can until they are either too breathless or can no longer keep up with the beeps at which time the test ends. It is reported as the distance walked. The ISWT is found to be valid, reliable and responsive in patients with chronic heart and respiratory disease (39-41).

For peer review only

Heart rate, oxygen saturations, blood pressure and BORG breathlessness score will be recorded before and after completion of the walk tests. A practice walk test will be performed as per the guidelines for this exercise test.

Frailty

Fried's frailty definition will be recorded, which is based on patient reported weight loss and exhaustion along with measured slowness (gait speed), weakness (hand grip) and physical activity (42). This has been shown to provide a standardised definition of frailty with predictive validity in the community dwelling older population (43). The Rockwood Frailty Scale will also be recorded. This is completed by the researcher in response to medical history and outcome measures taken during the visit and has also been demonstrated as a valid and reliable way of documenting frailty (44).

Participants will also complete the Timed Up and Go test where the patient starts in a seated position, stands and walks 3 metres, then turns around and returns to the seated position (45). The patient is timed how long this process takes.

The Short Physical Performance Battery (46) which includes the Four Metre Gait Speed (4MGS) test and the Sit-to-Stand test and assessing balance with the patient standing in different positions (side by side stand, semi tandem stand, tandem stand) will be completed, along with handgrip and quadriceps strength.

Health Economic Modelling

The feasibility study is based on the future RCT estimating the lifetime incremental cost per QALY gained. The objectives are to identify: the main NHS and prescribed specialist services (PSS) cost components; the resource use and unit cost data required for each of these components; potential

For peer review only

For peer review only - http://bmjopen.bmj.com/site/about/guidelines.xhtml

sources of HRQOL data suitable for estimating QALYs in this patient group; potential sources that could be used to estimate residual life expectancy and other long-term outcomes among patients.

Semi-structured interviews.

Audio-recorded interviews will be conducted privately face-to-face or via telephone between the participant and an interviewer, following informed consent. Interviews will take place with patients and GPs until data saturation is perceived. The interviews are anticipated to be between 30 minutes and one hour duration and will be professionally transcribed verbatim, with identifiable information removed. The transcription will be performed by an external company and a confidentiality agreement will be in place. Interview prompts will be devised based upon relevant literature, experience of the team and consultation with patient representatives.

Patients consented for the feasibility trial who are willing and able, and healthcare staff from the participating practices, will be interviewed.

The interviews will explore patients' experience of breathlessness, taking part in the trial, and their related healthcare. Patients will also be asked about the acceptability of the research visits and outcome measures performed and about their understanding of the trial. Interviews with healthcare staff will seek to understand what is usual care and any influence that taking part in the trial has on usual care. The clinician interviews will include questions about what a diagnostic pathway should or could look like from the perspective of the health professionals. They will also explore any barriers to screening patients for eligibility, challenges in implementing the pathway, or perceived benefits of the intervention.

For peer review only - http://bmjopen.bmj.com/site/about/guidelines.xhtml

Data Management

Paper based anonymised study records will be stored in locked filing cabinets within a locked office at Glenfield Hospital. Electronic records will be stored on a restricted access, secure University of Leicester and University Hospitals of Leicester NHS Trust computer system, maintained by the Trust. Audio recordings will be done using an encrypted Dictaphone. The recordings will be uploaded to secure files on University of Leicester and University Hospitals of Leicester computers then deleted from the Dictaphone. Access to the files will be restricted and password protected.

Data Analysis

Data analysis will be performed in an exploratory fashion. Descriptive statistics, number and percentage for categorical data and mean and standard deviation or median and inter-quartile range for non-normally distributed continuous data will be present for all demographics, baseline characteristics and questionnaire scores. Normality of the baseline characteristics will be determined using the Kolmogorov-Smirnov test (KS-test) or Shapiro-Wilkes test depending on final recruitment numbers.

SPSS version 26 will be used for statistical analysis. Graphpad software will be used for any figures.

Data analysis will be performed on the complete dataset utilising all participants.

Secondary outcomes for both groups will be described as mean (SD) and median [IQR] for normally or non-normally distributed data respectively. The time to diagnosis will be analysed using survival analyses based on Cox proportional hazards survival modelling. The proportion of patients with valid diagnosis at three months and one year will be described and compared using chi squared tests.

For peer review only - http://bmjopen.bmj.com/site/about/guidelines.xhtml

Qualitative Analysis

The interviews will be reviewed using thematic analysis (47), supported by NVivo software. This approach follows six distinct stages: familiarisation with data; generating initial codes; searching for themes; reviewing themes; defining and naming themes and producing the report (48). Initial coding will be carried out and a sample of interviews will be coded by a second member of the team to ensure consistency and to enhance interpretive authenticity. Throughout the data analysis, an iterative approach will be undertaken with the research team meeting to discuss and review emerging themes and search for accounts that provide contesting views of the same phenomena or identify different phenomena. Analysis will continue until data saturation and themes will be synthesised and supported by using relevant quotes from the data. Patient representatives will be invited to comment on the emerging themes from the patient interviews to assess whether important issues may have been missed which could be included in subsequent interviews.

Protocol amendments

Any changes to the study protocol outlined in this paper will be approved by Nottingham 1 Research Ethics Committee. This will be in agreement with Sponsor University of Leicester and University Hospitals of Leicester Research department.

Ethics and Dissemination

Ethical Approval: The Research Ethics Committee Nottingham 1 have provided ethical approval for this research study (REC Reference: 19/EM/0201).

For peer review only - http://bmjopen.bmj.com/site/about/guidelines.xhtml

Monitoring

A Trial Steering Committee (TSC) will be convened to provide oversight and support to the project. The committee will comprise of an independent Chair, independent members including clinicians, experts in breathlessness, statistician and policy experts, patient representative members and the Principal Investigator (PI). The Trial Co-ordinator and will attend meetings as appropriate. A TSC Charter will be put in place and 'Conflict of Interest' declarations obtained for all members and attendees. The TSC will meet as required to monitor the progress of the study, adherence to the protocol, progress of the study, consideration of new information of relevance to the research question and participant safety.

A Trial Management Group (TMG) has been established during the preparation of the study. Group members include the PI, Research Associate/Project lead, Trial Co-ordinator and Research Assistants. Other collaborators and Leicester Clinical Trials Unit (LCTU), specialities with specific expertise will attend as appropriate. The TMG will be held at least monthly to monitor all aspects of the conduct and progress of the study, ensure that the protocol is adhered to and take appropriate action to safeguard participants and the quality of the study itself.

Dissemination

Results from the study will be disseminated by presentations at relevant meetings and conferences including British Thoracic Society (BTS) and Primary Care Respiratory Society (PCRS), as well as by peer-reviewed publications and through patient presentations and newsletters to patients, where available. The results will also be shared with local primary and secondary care partners. Following

For peer review only

the feasibility trial, the aim is to conduct a national multicentre trial to assess clinical and cost effectiveness of a diagnostic pathway for breathlessness. The feasibility outcomes collected and qualitative analysis will help refine the design of a future trial.

Funding statement: This work was funded by a NIHR Clinician Scientist Fellowship (CS-2016-16-020) awarded to Dr Rachael A Evans. Professor Natalie Armstrong is supported by a Health Foundation Improvement Science Fellowship and also by the National Institute for Health Research (NIHR) Applied Research Collaboration East Midlands (ARC EM). The views expressed are those of the authors and not necessarily those of the National Health Service, the NIHR, or the Department of Health.

Competing interests: The authors declare no competing interests.

Authors' contributions: Dr Rachael Evans conceived the research idea, and developed the theory and plan for this study. Ms Gillian Doe drafted the initial manuscript. Mr Simon Wathall developed the Electronic template for use in the recruitment strategy. All authors reviewed, commented and approved the manuscript.

Acknowledgements: We acknowledge and are grateful to our public and patient involvement (PPI) members; Winifred Smart, Jagruti Lalseta, Brian Davies, Paul Ashby and Tony Watling. RAE thanks Professor Mike Morgan and the Research Design Service, University of Leicester for their support with the NIHR clinician scientist fellowship application. We also thank the clinicians who contributed to the development of the structured diagnostic pathway including Professor Ruth Green, Mrs Louise Clayton, Mrs Karen Moore, Mr Alex Woodward, Mrs Jo Szymkowiak, Ms Alison

For peer review only - http://bmjopen.bmj.com/site/about/guidelines.xhtml

Scott, Ms Jane Giles. The work is supported by the NIHR Leicester Biomedical Research Centre-Respiratory.

For peer review only

For peer review only - http://bmjopen.bmj.com/site/about/guidelines.xhtml

References

1. Hutchinson A, Pickering A, Williams P et al. Breathlessness and presentation to the emergency department: a survey and clinical record review. *BMC Pulm Med* 2017;17(1):53.
2. Laribi S, Keijzers G, van Meer O, et al. Epidemiology of patients presenting with dyspnea to emergency departments in Europe and the Asia-Pacific region. *European Journal of Emergency Medicine*2019;26(5):345-9.
3. Frese T, Sobeck C, Herrmann K et al. Dyspnea as the reason for encounter in general practice. *J Clin Med Res* 2011;3(5):239-46.
4. Stevens JP, Dechen T, Schwartzstein R et al. Prevalence of Dyspnea Among Hospitalized Patients at the Time of Admission. *Journal of pain and symptom management* 2018;56(1):15-22.e2.
5. Currow DC, Plummer JL, Crockett A et al.. A community population survey of prevalence and severity of dyspnea in adults. *J Pain Symptom Manage* 2009;38(4):533-45.
6. Bowden JA, To TH, Abernethy AP et al. Predictors of chronic breathlessness: a large population study. *BMC Public Health* 2011;11:33.
7. Gronseth R, Vollmer WM, Hardie JA et al. Predictors of dyspnoea prevalence: results from the BOLD study. *Eur Respir J* 2014;43(6):1610-20.
8. van Mourik Y, Rutten FH, Moons KG et al. Prevalence and underlying causes of dyspnoea in older people: a systematic review. *Age Ageing* 2014;43(3):319-26.
9. Smith AK, Currow DC, Abernethy APet al. Prevalence and Outcomes of Breathlessness in Older Adults: A National Population Study. *Journal of the American Geriatrics Society* 2016;64(10):2035-41.
10. Frostad A, Soyseth V, Andersen A et al. Respiratory symptoms as predictors of all-cause mortality in an urban community: a 30-year follow-up. *J Intern Med* 2006;259(5):520-9.
11. Gillespie DJ, Staats BA. Unexplained dyspnea. *Mayo Clin Proc* 1994;69(7):657-63.
12. IMPRESS. Breathlessness IMPRESS Tips (BITs) For clinicians 2016 [Available from: https://www.respiratoryfutures.org.uk/resources/impress-documents/impress-breathlessness-algorithm/.
13. Sandberg J, Ekstrom M, Borjesson M et al. Underlying contributing conditions to breathlessness among middle-aged individuals in the general population: a cross-sectional study. *BMJ Open Respir Res* 2020;7(1).
14. NICE. Clinical Knowledge Summary on Breathlessness 2017 [Available from: https://cks.nice.org.uk/breathlessness.
15. Jones RC, Price D, Ryan D et al. Opportunities to diagnose chronic obstructive pulmonary disease in routine care in the UK: a retrospective study of a clinical cohort. *Lancet Respir Med* 2014;2(4):267-76.
16. Bottle A, Kim D, Aylin P et al. Routes to diagnosis of heart failure: observational study using linked data in England. *Heart* 2018;104(7):600-5.
17. Collard HR, Tino G, Noble PW et al. Patient experiences with pulmonary fibrosis. *Respir Med* 2007;101(6):1350-4.
18. du Bois RM. An earlier and more confident diagnosis of idiopathic pulmonary fibrosis. *Eur Respir Rev*2012;21(124):141-6.
19. Aaron SD, Boulet LP, Reddel HK et al. Underdiagnosis and Overdiagnosis of Asthma. *American Journal of Respiratory and Critical Care Medicine* 2018;198(8):1012-20.
20. Heffler E, Crimi C, Mancuso S et al. Misdiagnosis of asthma and COPD and underuse of spirometry in primary care unselected patients. *Respir Med* 2018;142:48-52.
21. Royal College of Physicians. National Asthma and COPD Audit Programme (NACAP): primary care workstream. Wales primary care clinical audit report 2020.: RCP; 2020.
22. Singh H, Dickinson JA, Theriault G et al. Overdiagnosis: causes and consequences in primary health care. *Can Fam Physician* 2018;64(9):654-9.

For peer review only - http://bmjopen.bmj.com/site/about/guidelines.xhtml

23. Medical Research Council. Developing and evaluating complex interventions. 2019.
24. Listening into Action 2019 [Available from: http://www.listeningintoaction.co.uk/LiA-info/index.php.
25. Ternovski J. STRATARAND: Stata module to perform stratified random assignment. 2015.
26. Foster N, Mullis R, Hill J et al. Effect of Stratified Care for Low Back Pain in Family Practice (IMPaCT Back): A Prospective Population-Based Sequential Comparison. *The Annals of Family Medicine* 2014;12:102-11.
27. Kroenke K, Spitzer RL, Williams JB et al. An ultra-brief screening scale for anxiety and depression: the PHQ-4. *Psychosomatics* 2009;50(6):613-21.
28. Department of Health and Social Care. General Practice Physical Activity Questionnaire. 2013.
29. Evans RA, Singh SJ, Williams JE et al. The development of a self-reported version of the chronic heart questionnaire. *J Cardiopulm Rehabil Prev* 2011;31(6):365-72.
30. (5Q5D5L). E. Euroqol (5Q5D-5L) 2016 [Available from: www.euroqol.org/eq-5d-products/eq-5d-5l.html .
31. Meek PM, Banzett R, Parsall MB et al. Reliability and Validity of the Multidimensional Dyspnea Profile. *Chest* 2012;141(6):1546-53.
32. Witek TJ, Jr., Mahler DA. Minimal important difference of the transition dyspnoea index in a multinational clinical trial. *Eur Respir J* 2003;21(2):267-72.
33. Fletcher CM, Elmes PC, Fairbairn AS et al. The significance of respiratory symptoms and the diagnosis of chronic bronchitis in a working population. *Br Med J* 1959;2(5147):257-66.
34. Zigmond AS, Snaith RP. The hospital anxiety and depression scale. *Acta Psychiatr Scand* 1983;67(6):361-70.
35. Hibbard JH, Mahoney ER, Stockard J et al. Development and testing of a short form of the patient activation measure. *Health Serv Res* 2005;40(6 Pt 1):1918-30.
36. Actigraph. [Available from: https://actigraphcorp.com/support/activity-monitors/gt3x/
37. GeneActiv. [Available from: https://www.activinsights.com/actigraphy/geneactiv-original/
38. Singh SJ, Morgan MD, Scott S et al. Development of a shuttle walking test of disability in patients with chronic airways obstruction. *Thorax* 1992;47(12):1019-24.
39. Singh SJ, Puhan MA, Andrianopoulos V et al. An official systematic review of the European Respiratory Society/American Thoracic Society: measurement properties of field walking tests in chronic respiratory disease. *Eur Respir J* 2014;44(6):1447-78.
40. Holland AE, Spruit MA, Troosters T et al. An official European Respiratory Society/American Thoracic Society technical standard: field walking tests in chronic respiratory disease. *Eur Respir J* 2014;44(6):1428-46.
41. Morales FJ, Martínez A, Méndez M, et al. A shuttle walk test for assessment of functional capacity in chronic heart failure. *Am Heart J* 1999;138(2 Pt 1):291-8.
42. Fried LP, Tangen CM, Walston J et al. Frailty in older adults: evidence for a phenotype. *J Gerontol A Biol Sci Med Sci* 2001;56(3):M146-56.
43. Newman AB, Hirsch C, Tangen CM et al. Frailty in Older Adults: Evidence for a Phenotype. *The Journals of Gerontology: Series A* 2001;56(3):M146-M57.
44. Rockwood K, Song X, MacKnight C et al. A global clinical measure of fitness and frailty in elderly people. *CMAJ* 2005;173(5):489-95.
45. Podsiadlo D, Richardson S. The timed "Up & Go": a test of basic functional mobility for frail elderly persons. *J Am Geriatr Soc*1991;39(2):142-8.
46. Guralnik JM, Simonsick EM, Ferrucci L et al. A short physical performance battery assessing lower extremity function: association with self-reported disability and prediction of mortality and nursing home admission. *J Gerontol* 1994;49(2):M85-94.
47. Braun V, Clarke V. Reflecting on reflexive thematic analysis. *Qual Res Sport Exerc* 2019;11(4):589-97.
48. Clarke V, Braun V. Thematic analysis. *J Posit Psychol* 2017;12(3):297-8.

For peer review only - http://bmjopen.bmj.com/site/about/guidelines.xhtml

Figure 1. The electronic template triggered on patient electronic healthcare record

Figure 2. Study Schedule

Figure 3. Diagnostic Pathway

For peer review only

For peer review only - http://bmjopen.bmj.com/site/about/guidelines.xhtml

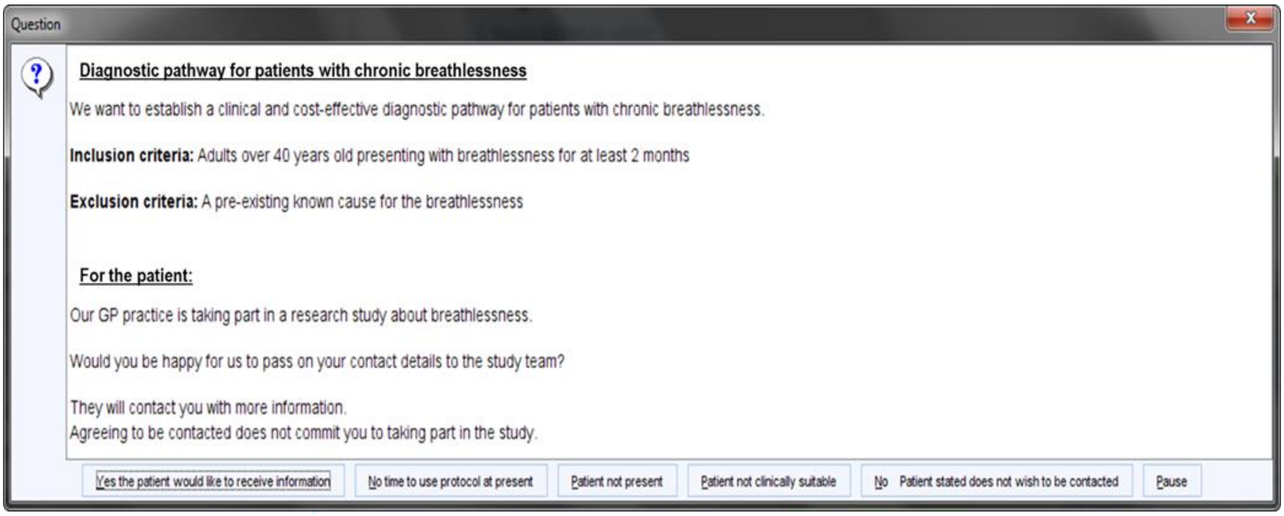
Question

Diagnostic pathway for patients with chronic breathlessness

We want to establish a clinical and cost-effective diagnostic pathway for patients with chronic breathlessness.

Inclusion criteria: Adults over 40 years old presenting with breathlessness for at least 2 months

Exclusion criteria: A pre-existing known cause for the breathlessness

For the patient:

Our GP practice is taking part in a research study about breathlessness.

Would you be happy for us to pass on your contact details to the study team?

They will contact you with more information.
Agreeing to be contacted does not commit you to taking part in the study.

Yes the patient would like to receive information | No time to use protocol at present | Patient not present | Patient not clinically suitable | No Patient stated does not wish to be contacted | Pause

For peer review only

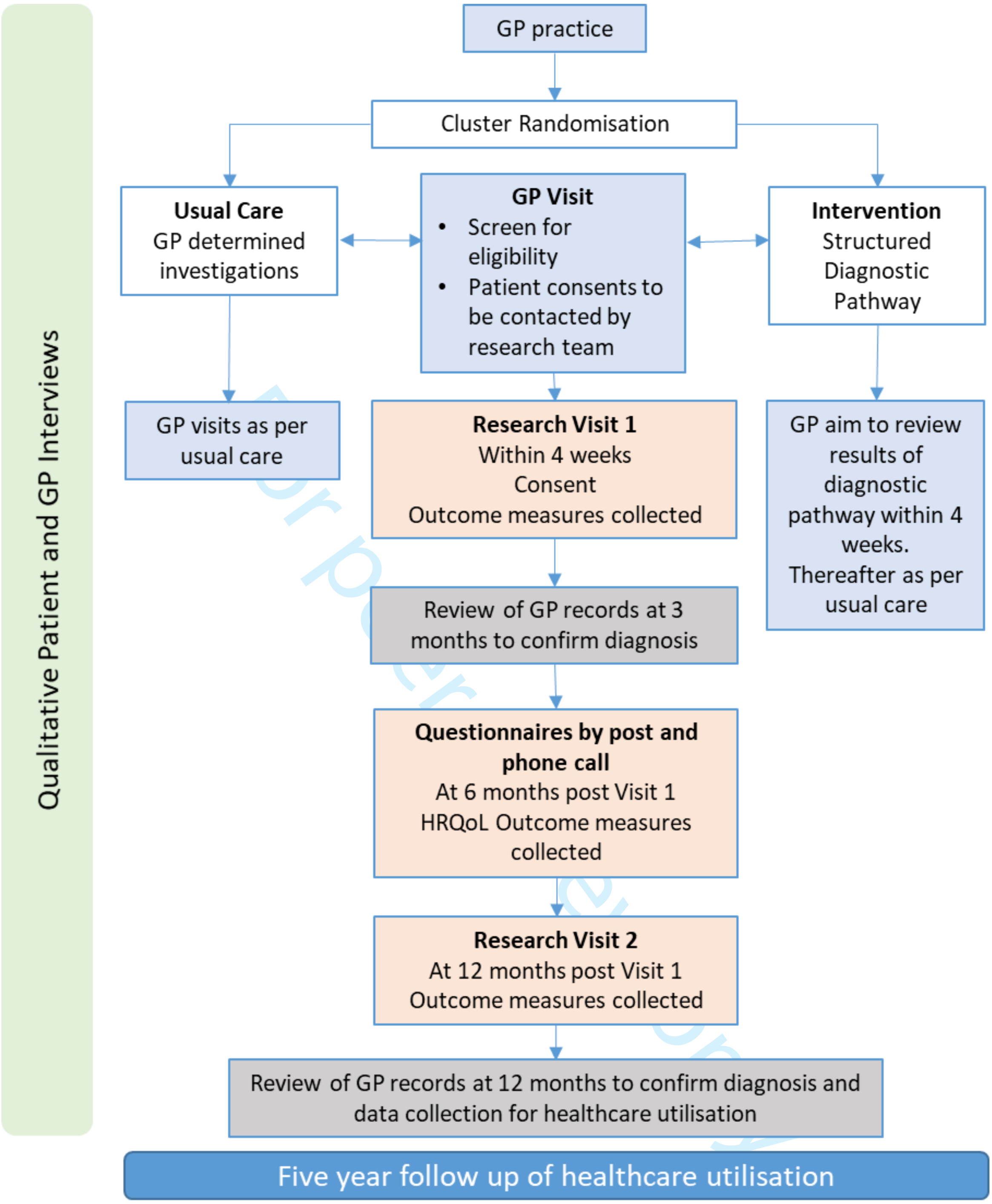
Qualitative Patient and GP Interviews
GP practice
Cluster Randomisation
Usual Care
GP determined investigations
GP Visit
Screen for eligibility
Patient consents to be contacted by research team
Intervention
Structured Diagnostic Pathway
GP visits as per usual care
Research Visit 1
Within 4 weeks
Consent
Outcome measures collected
GP aim to review results of diagnostic pathway within 4 weeks.
Thereafter as per usual care
Review of GP records at 3 months to confirm diagnosis
Questionnaires by post and phone call
At 6 months post Visit 1
HRQoL Outcome measures collected
Research Visit 2
At 12 months post Visit 1
Outcome measures collected
Review of GP records at 12 months to confirm diagnosis and data collection for healthcare utilisation
Five year follow up of healthcare utilisation

FUNDED BY NIHR | National Institute for Health Research

UNIVERSITY OF LEICESTER

NHS University Hospitals of Leicester NHS Trust

Breathlessness Diagnostic Pathway

Diagnostic pathway for initial presentation of chronic breathlessness

For adults > 40 yrs old presenting with breathlessness for > 2 months please follow this pathway.

Please Read code for Breathlessness. For acute breathlessness, please follow usual procedure for assessment and action.

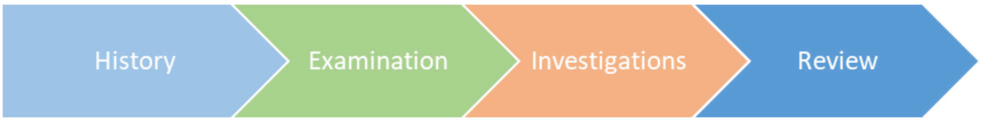

STEP 1. Focused History and Examination

As usual, please take a clear history to assess possible causes and impact of breathlessness. Please include the following:

HISTORY	EXAMINATION
✓ Onset and duration of breathlessness<br>✓ At rest/exertional<br>✓ Nature of breathlessness<br>✓ Aggravating and relieving factors<br>✓ Associated symptoms (e.g. chest pain, cough, wheeze, ankle swelling, palpitations)<br>✓ Orthopnoea, PND<br>✓ Levels of exercise and daily activity<br>✓ Impact on everyday life and MRC Dyspnoea scale<br>✓ Co-morbidities and medications<br>✓ Smoking history including pack years and substance smoked<br>✓ Environmental and occupational risk factors	✓ Vital signs: resting HR and rhythm, O_2 saturation, RR and BP<br>✓ Observe general appearance and breathing pattern (increase use of accessory muscles)<br>✓ Assess JVP<br>✓ Check for peripheral oedema<br>✓ Auscultate lungs (particularly for bi-basal crackles)<br>✓ Auscultate cardiac sounds (listen for murmurs including right carotid area for aortic stenosis)<br>✓ BMI (weight kg/height m^2)

STEP 2. Investigations for Chronic Breathlessness

Please initiate the investigations listed below to rule in/out common causes of breathlessness.

Investigation	Implications for diagnosis
**Body Mass Index (BMI)**	To diagnose obesity if >30kg/m^2
**ECG**	To detect arrhythmias or diagnoses suggestive of ischaemic heart disease
**Chest X-ray**	To detect signs of heart failure or pulmonary pathology e.g. pleural effusion, interstitial changes
**Spirometry** (If FEV_1/FEV_6 <0.75 perform full spirometry)	Obstructive pattern (FEV_1/FVC < 0.70) may suggest airway disease such as COPD or asthma<br>Restrictive pattern (FEV_1/FVC > 0.70 but FEV_1 and FVC < 80% predicted) may suggest conditions such as obesity, interstitial lung disease, chest wall abnormality or neuromuscular disease
**FBC**	Haemoglobin to exclude anaemia<br>Eosinophil count if >0.3 x10^9/L can support a diagnosis of asthma
**BNP profile**	To exclude heart failure
**Urea and electrolytes/ TSH**	To exclude metabolic causes
**Activity Questionnaire GPPAQ**	To assess physical activity levels
**PHQ4**	Screening questionnaires for symptoms of anxiety and depression

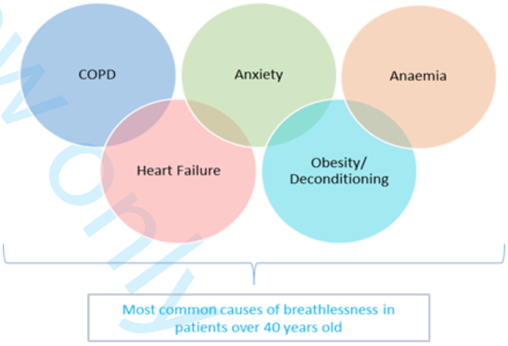

MRC Grade	Degree of breathlessness related to activity
1	Not troubled by breathlessness except on strenuous exercise
2	Short of breath when hurrying on level ground or when walking up a slight hill
3	Walks slower than contemporaries on level ground due to breathlessness, or has to stop for breath when walking at own pace
4	Stops for breath after walking about 100m or after a few minutes on level ground
5	Too breathless to leave the house, or breathless when dressing or undressing

STEP 3. Please aim to review results and patient within 1 month. Please use additional document for guidelines and onward referral as appropriate

For peer review only - http://bmjopen.bmj.com/site/about/guidelines.xhtml

Diagnostic Uncertainty → Please consider referral to Breathlessness Clinic (referral form in PRISM)

SPIRIT

STANDARD PROTOCOL ITEMS: RECOMMENDATIONS FOR INTERVENTIONAL TRIALS

SPIRIT 2013 Checklist: Recommended items to address in a clinical trial protocol and related documents*

Section/item	Item No	Description	Page number and details
**Administrative information**			
Title	1	Descriptive title identifying the study design, population, interventions, and, if applicable, trial acronym	1
Trial registration	2a	Trial identifier and registry name. If not yet registered, name of intended registry	3 ISRCTN14483247
	2b	All items from the World Health Organization Trial Registration Data Set	
Protocol version	3	Date and version identifier	Version 1 for publication.
Funding	4	Sources and types of financial, material, and other support	20 Funded by a NIHR Clinician Scientist Fellowship (CS-2016-16-020) awarded to Dr Rachael A Evans.
Roles and responsibilities	5a	Names, affiliations, and roles of protocol contributors	1
	5b	Name and contact information for the trial sponsor	7
	5c	Role of study sponsor and funders, if any, in study design; collection, management, analysis, and interpretation of data; writing of the report; and the decision to submit the report for publication, including whether they will have ultimate authority over any of these activities	12 and 18
	5d	Composition, roles, and responsibilities of the coordinating centre, steering committee, endpoint adjudication committee, data management team, and other individuals or groups overseeing the trial, if applicable (see Item 21a for data monitoring committee)	18 and 19

For peer review only

For peer review only - http://bmjopen.bmj.com/site/about/guidelines.xhtml

**Introduction**			
Background and rationale	6a	Description of research question and justification for undertaking the trial, including summary of relevant studies (published and unpublished) examining benefits and harms for each intervention	4 and 5
	6b	Explanation for choice of comparators	
Objectives	7	Specific objectives or hypotheses	5
Trial design	8	Description of trial design including type of trial (eg, parallel group, crossover, factorial, single group), allocation ratio, and framework (eg, superiority, equivalence, noninferiority, exploratory)	7<br>Feasibility cluster randomised controlled trial.
**Methods: Participants, interventions, and outcomes**			
Study setting	9	Description of study settings (eg, community clinic, academic hospital) and list of countries where data will be collected. Reference to where list of study sites can be obtained	9
Eligibility criteria	10	Inclusion and exclusion criteria for participants. If applicable, eligibility criteria for study centres and individuals who will perform the interventions (eg, surgeons, psychotherapists)	8
Interventions	11a	Interventions for each group with sufficient detail to allow replication, including how and when they will be administered	11
	11b	Criteria for discontinuing or modifying allocated interventions for a given trial participant (eg, drug dose change in response to harms, participant request, or improving/worsening disease)	N/A
	11c	Strategies to improve adherence to intervention protocols, and any procedures for monitoring adherence (eg, drug tablet return, laboratory tests)	8 and 9 (recruitment)<br>11
	11d	Relevant concomitant care and interventions that are permitted or prohibited during the trial	11
Outcomes	12	Primary, secondary, and other outcomes, including the specific measurement variable (eg, systolic blood pressure), analysis metric (eg, change from baseline, final value, time to event), method of aggregation (eg, median, proportion), and time point for each outcome. Explanation of the clinical relevance of chosen efficacy and harm outcomes is strongly recommended	11-16

For peer review only - http://bmjopen.bmj.com/site/about/guidelines.xhtml

Participant timeline	13	Time schedule of enrolment, interventions (including any run-ins and washouts), assessments, and visits for participants. A schematic diagram is highly recommended (see Figure)	Figure 2. Study Schedule diagram
Sample size	14	Estimated number of participants needed to achieve study objectives and how it was determined, including clinical and statistical assumptions supporting any sample size calculations	11<br>Primary outcome is recruitment rate for this feasibility trial so no formal sample size.
Recruitment	15	Strategies for achieving adequate participant enrolment to reach target sample size	8 and 9 (recruitment strategy)
**Methods: Assignment of interventions (for controlled trials)**			
Allocation:			
Sequence generation	16a	Method of generating the allocation sequence (eg, computer-generated random numbers), and list of any factors for stratification. To reduce predictability of a random sequence, details of any planned restriction (eg, blocking) should be provided in a separate document that is unavailable to those who enrol participants or assign interventions	6 (randomisation of GP practices)
Allocation concealment mechanism	16b	Mechanism of implementing the allocation sequence (eg, central telephone; sequentially numbered, opaque, sealed envelopes), describing any steps to conceal the sequence until interventions are assigned	8<br>The GP practices will be randomised 1:1 and stratified by CCG using Statarand (25), a Stata randomisation module (Boston College Department of Economics).
Implementation	16c	Who will generate the allocation sequence, who will enrol participants, and who will assign participants to interventions	7 and 8<br>Cluster randomised at level of GP practice.
Blinding (masking)	17a	Who will be blinded after assignment to interventions (eg, trial participants, care providers, outcome assessors, data analysts), and how	N/A
	17b	If blinded, circumstances under which unblinding is permissible, and procedure for revealing a participant's allocated intervention during the trial	N/A

For peer review only - http://bmjopen.bmj.com/site/about/guidelines.xhtml

**Methods: Data collection, management, and analysis**			
Data collection methods	18a	Plans for assessment and collection of outcome, baseline, and other trial data, including any related processes to promote data quality (eg, duplicate measurements, training of assessors) and a description of study instruments (eg, questionnaires, laboratory tests) along with their reliability and validity, if known. Reference to where data collection forms can be found, if not in the protocol	12
	18b	Plans to promote participant retention and complete follow-up, including list of any outcome data to be collected for participants who discontinue or deviate from intervention protocols	5<br>One of the feasibility aims is to assess retention rate.
Data management	19	Plans for data entry, coding, security, and storage, including any related processes to promote data quality (eg, double data entry; range checks for data values). Reference to where details of data management procedures can be found, if not in the protocol	17<br>Also detailed in the PIS (uploaded as supplementary material)
Statistical methods	20a	Statistical methods for analysing primary and secondary outcomes. Reference to where other details of the statistical analysis plan can be found, if not in the protocol	17 and 18
	20b	Methods for any additional analyses (eg, subgroup and adjusted analyses)	
	20c	Definition of analysis population relating to protocol non-adherence (eg, as randomised analysis), and any statistical methods to handle missing data (eg, multiple imputation)	
**Methods: Monitoring**			
Data monitoring	21a	Composition of data monitoring committee (DMC); summary of its role and reporting structure; statement of whether it is independent from the sponsor and competing interests; and reference to where further details about its charter can be found, if not in the protocol. Alternatively, an explanation of why a DMC is not needed	Data will be monitored, checked and cleaned by the study team for this feasibility study.
	21b	Description of any interim analyses and stopping guidelines, including who will have access to these interim results and make the final decision to terminate the trial	
Harms	22	Plans for collecting, assessing, reporting, and managing solicited and spontaneously reported adverse events and other unintended effects of trial interventions or trial conduct	9 and 10

For peer review only - http://bmjopen.bmj.com/site/about/guidelines.xhtml

Auditing	23	Frequency and procedures for auditing trial conduct, if any, and whether the process will be independent from investigators and the sponsor	The University of Leicester, as Sponsor, operates a risk based monitoring and audit programme, to which this study will be subject.
**Ethics and dissemination**			
Research ethics approval	24	Plans for seeking research ethics committee/institutional review board (REC/IRB) approval	3<br>The Research Ethics Committee Nottingham 1 has provided ethical approval for this research study (REC Reference: 19/EM/0201).
Protocol amendments	25	Plans for communicating important protocol modifications (eg, changes to eligibility criteria, outcomes, analyses) to relevant parties (eg, investigators, REC/IRBs, trial participants, trial registries, journals, regulators)	18
Consent or assent	26a	Who will obtain informed consent or assent from potential trial participants or authorised surrogates, and how (see Item 32)	10<br>Informed consent will be obtained from members of the study team.
	26b	Additional consent provisions for collection and use of participant data and biological specimens in ancillary studies, if applicable	N/A
Confidentiality	27	How personal information about potential and enrolled participants will be collected, shared, and maintained in order to protect confidentiality before, during, and after the trial	12 and 17
Declaration of interests	28	Financial and other competing interests for principal investigators for the overall trial and each study site	20<br>Authors declare no competing interests.
Access to data	29	Statement of who will have access to the final trial dataset, and disclosure of contractual agreements that limit such access for investigators	Detailed in the PIS uploaded as supplementary material.

For peer review only

For peer review only - http://bmjopen.bmj.com/site/about/guidelines.xhtml

Ancillary and post-trial care	30	Provisions, if any, for ancillary and post-trial care, and for compensation to those who suffer harm from trial participation	9<br>Participation is considered to be very low risk.
Dissemination policy	31a	Plans for investigators and sponsor to communicate trial results to participants, healthcare professionals, the public, and other relevant groups (eg, via publication, reporting in results databases, or other data sharing arrangements), including any publication restrictions	19-20
	31b	Authorship eligibility guidelines and any intended use of professional writers	
	31c	Plans, if any, for granting public access to the full protocol, participant-level dataset, and statistical code	
**Appendices**			
Informed consent materials	32	Model consent form and other related documentation given to participants and authorised surrogates	Uploaded Appendix A and B
Biological specimens	33	Plans for collection, laboratory evaluation, and storage of biological specimens for genetic or molecular analysis in the current trial and for future use in ancillary studies, if applicable	N/A

*It is strongly recommended that this checklist be read in conjunction with the SPIRIT 2013 Explanation & Elaboration for important clarification on the items. Amendments to the protocol should be tracked and dated. The SPIRIT checklist is copyrighted by the SPIRIT Group under the Creative Commons "Attribution-NonCommercial-NoDerivs 3.0 Unported" license.

For peer review only

For peer review only - http://bmjopen.bmj.com/site/about/guidelines.xhtml

BMJ Open

A protocol for a feasibility study of a multi-centre cluster randomised control trial to investigate the clinical and cost effectiveness of a structured diagnostic pathway in primary care for chronic breathlessness.

Journal:	*BMJ Open*
Manuscript ID	bmjopen-2021-057362.R1
Article Type:	Protocol
Date Submitted by the Author:	13-Oct-2021
Complete List of Authors:	Doe, Gillian; University of Leicester, Respiratory Sciences<br>Clanchy, Jill; University of Leicester<br>Wathall, Simon; Keele University, Institute of Primary Care & Health Sciences<br>Chantrell, Stacey; University Hospitals of Leicester NHS Trust<br>Edwards, Sarah; University Hospitals of Leicester NHS Trust<br>Baxter, Noel; International Primary Care Respiratory Group<br>Jackson, Darren; Barwell & Hollycroft Medical Centres<br>Armstrong, Natalie ; University of Leicester, Health Sciences<br>Steiner, Michael; Institute for Lung Health, NIHR Leicester Biomedical Research Centre – Respiratory, University of Leicester, Glenfield Hospital,<br>Evans , Rachael A; University of Leicester, Department of Respiratory Science; University Hospitals of Leicester NHS Trust
**Primary Subject Heading**:	Health services research
Secondary Subject Heading:	General practice / Family practice, Qualitative research, Respiratory medicine
Keywords:	PRIMARY CARE, RESPIRATORY MEDICINE (see Thoracic Medicine), QUALITATIVE RESEARCH, Quality in health care < HEALTH SERVICES ADMINISTRATION & MANAGEMENT

SCHOLARONE™
Manuscripts

I, the Submitting Author has the right to grant and does grant on behalf of all authors of the Work (as defined in the below author licence), an exclusive licence and/or a non-exclusive licence for contributions from authors who are: i) UK Crown employees; ii) where BMJ has agreed a CC-BY licence shall apply, and/or iii) in accordance with the terms applicable for US Federal Government officers or employees acting as part of their official duties; on a worldwide, perpetual, irrevocable, royalty-free basis to BMJ Publishing Group Ltd ("BMJ") its licensees and where the relevant Journal is co-owned by BMJ to the co-owners of the Journal, to publish the Work in this journal and any other BMJ products and to exploit all rights, as set out in our licence.

The Submitting Author accepts and understands that any supply made under these terms is made by BMJ to the Submitting Author unless you are acting as an employee on behalf of your employer or a postgraduate student of an affiliated institution which is paying any applicable article publishing charge ("APC") for Open Access articles. Where the Submitting Author wishes to make the Work available on an Open Access basis (and intends to pay the relevant APC), the terms of reuse of such Open Access shall be governed by a Creative Commons licence – details of these licences and which Creative Commons licence will apply to this Work are set out in our licence referred to above.

Other than as permitted in any relevant BMJ Author's Self Archiving Policies, I confirm this Work has not been accepted for publication elsewhere, is not being considered for publication elsewhere and does not duplicate material already published. I confirm all authors consent to publication of this Work and authorise the granting of this licence.

For peer review only

Word Count = 3853

Title: A protocol for a feasibility study of a multi-centre cluster randomised control trial to investigate the clinical and cost effectiveness of a structured diagnostic pathway in primary care for chronic breathlessness.

(Breathe DEEP: Breathlessness – DiagnosE Early in Primary Care.)

Authors:

Ms Gillian Doe[1], Ms Jill Clanchy[2], Mr Simon Wathall[3], Ms Stacey Chantrell[4], Ms Sarah Edwards[4], Dr Noel Baxter[5], Dr Darren Jackson[6], Prof Natalie Armstrong[7], Prof Michael Steiner[1,4] and *Dr Rachael A Evans[1,4]

Institutions:

1. Department of Respiratory Science, University of Leicester, Leicester, UK.

2. Clinical Trials Unit, University of Leicester, Leicester, UK.

3. Clinical Trials Unit, Keele University, UK.

4. NIHR Biomedical Research Centre – Respiratory theme, University Hospitals of Leicester NHS Trust, Leicester, UK.

5. International Primary Care Respiratory Group, UK.

6. Barwell & Hollycroft Medical Centres, Chair Hinckley & Bosworth Medical Alliance.

7. Department of Health Sciences, University of Leicester, Leicester, UK.

For peer review only

For peer review only - http://bmjopen.bmj.com/site/about/guidelines.xhtml

Author for correspondence: *Dr Rachael Evans, Leicester NIHR Biomedical Research Centre – Respiratory, Glenfield Hospital, Leicester, LE3 9QP (Email: re66@leicester.ac.uk). ORCID ID: https://orcid.org/0000-0002-1667-868X

Abstract

Introduction: Chronic breathlessness is a common and debilitating symptom, associated with high healthcare use and reduced quality of life. Challenges and delays in diagnosis for people with chronic breathlessness frequently occur, leading to delayed access to therapies. The overarching hypothesis is a symptom-based approach to diagnosis in primary care would lead to earlier diagnosis, and therefore earlier treatment and improved longer term outcomes including health-related quality of life.

This study aims to establish the feasibility of a multicentre cluster randomised controlled trial to assess the clinical and cost effectiveness of a structured diagnostic pathway for breathlessness in primary care.

Methods and Analysis: Ten General Practitioner (GP) practices across Leicester and Leicestershire will be cluster randomised to either a structured diagnostic pathway (intervention) or usual care. The structured diagnostic pathway includes a panel of investigations within one month. Usual care will proceed with patient care as per normal practice. Eligibility criteria include patients presenting with chronic breathlessness for the first time, who are over 40 years old and without a pre-existing diagnosis for their symptoms. An electronic template triggered at the point of consultation with the GP will aid opportunistic recruitment in primary care.

For peer review only - http://bmjopen.bmj.com/site/about/guidelines.xhtml

The primary outcome for this feasibility study is recruitment rate. Secondary outcome measures, including time to diagnosis, will be collected to help inform outcomes for the future trial and to assess the impact of an earlier diagnosis. These will include symptoms, health-related quality of life, exercise capacity, measures of frailty, physical activity and healthcare utilisation.

The study will include nested qualitative interviews with patients and healthcare staff to understand the feasibility outcomes, explore what is 'usual care' and the study experience.

Ethics and Dissemination: The Research Ethics Committee Nottingham 1 has provided ethical approval for this research study (REC Reference: 19/EM/0201).

Results from the study will be disseminated by presentations at relevant meetings and conferences including British Thoracic Society (BTS) and Primary Care Respiratory Society (PCRS), as well as by peer-reviewed publications and through patient presentations and newsletters to patients, where available.

Trial registration: ISRCTN14483247

Strengths and Limitations:

- A mixed methods approach will be used to both understand how breathlessness is perceived and diagnosed in primary care, and further interpret the findings of the feasibility study.
- The structured diagnostic pathway uses existing investigations available in primary care which will aid future implementation.
- The trial is embedded within clinical care and utilises opportunistic recruitment when patients present with breathlessness in primary care.
- The study design is a cluster randomised trial to minimise potential bias and contamination.

For peer review only

For peer review only - http://bmjopen.bmj.com/site/about/guidelines.xhtml

- The trial will be conducted in a single region in the UK and may limit the generalisability of the study findings

Introduction

Breathlessness is associated with high healthcare use, accounting for 5% of presentations to the emergency department (1, 2), approximately 4% of GP consultations (3) and reported by patients in 12% of medical admissions (4). Breathlessness is reported by around 9-11% (5, 6) of the general population, varying with severity, socioeconomic status (6, 7) and increasing with age to 25% in people over seventy years old (8, 9). Functional impairment from breathlessness, measured using the Medical Research Council (MRC) dyspnoea scale, is associated with reduced survival regardless of underlying diagnosis (10).

Two-thirds of breathlessness is caused by cardiorespiratory conditions (11). Clinical data shows that for patients over the age of forty the most common causes of breathlessness are Chronic Obstructive Pulmonary Disease (COPD), heart failure (HF), obesity, anaemia and anxiety (12, 13). These conditions can be potentially diagnosed or excluded using investigations that can be performed in community settings (12). All of these investigations are recommended in the National Institute for Health and Care Excellence (NICE) clinical knowledge summary (14) but there is no specific guidance on the timeframe within which they should be performed.

Primary care data have highlighted many missed opportunities over many years to diagnose conditions associated with breathlessness, such as COPD and HF (15, 16), with many patients being diagnosed only when the disease is severe or requiring hospitalisation (15, 16). These data indicate significant challenges in the deployment of simple diagnostics in the primary care setting. There

For peer review only

are also well-documented misdiagnoses for COPD, asthma and interstitial Lung Disease across healthcare settings (15, 17-21).

Our overarching hypothesis is that a symptom-based approach for diagnosis in primary care for patients with chronic breathlessness will lead to earlier diagnosis, earlier treatment and improved outcomes such as health-related quality of life. However, it is also important to consider the implications of over investigating and 'over-diagnosis' in patients and find the balance between clinical and cost effectiveness for a diagnostic pathway (22). A large and potentially expensive multi-centre cluster randomised controlled trial would be necessary to understand the clinical and cost effectiveness of a structured diagnostic pathway for chronic breathlessness. The scope of this study is to assess the feasibility of such a trial and help inform the design.

For this feasibility study the specific aims are:

1. To assess feasibility by recruitment and retention rate of patients in the trial to enable calculation of the number of GP practices, cluster sizes and duration of the ultimate RCT (Table 1).

2. To better understand 'usual care' through prospective observation and qualitative analysis, and to understand any influence of the trial design on usual care.

3. To determine the proposed primary outcome measure for the future trial and to increase understanding of what is an important and realistic difference whilst exploring potential of other outcome measures (Table 2).

4. Identify sources of data and how best to collect these in order to plan the economic evaluation that would accompany a full trial.

For peer review only

For peer review only - http://bmjopen.bmj.com/site/about/guidelines.xhtml

Table 1. Feasibility measures

Feasibility measures
Number of patients recruited per week per GP practice population size
Number of participating GP practices verses the number approached
Time for GPs to screen for eligibility
Number of eligible patients who agree to be approached by the research team verses total number of eligible patients
Number and timing of investigations in the diagnostic pathway completed
Acceptability of the research visit to the participants
Data collected from Interviews regarding participant experience of the trial
Data collected from Interviews regarding GP experience of participating in the trial and influence on their practice

Table 2. Secondary outcome measures

Secondary Outcome measures	Measurement Tool
Proportion of diagnoses in Usual care and Intervention within one year of presentation	Review of healthcare records for all participants
Time to diagnosis	Review of healthcare records for symptom presentation and diagnosis date
Health-related quality of life	Chronic Heart Questionnaire (CHQ)<br>Euroqol 5 Dimension 5 Level (EQ5D-5L)
Breathlessness	Dyspnoea -12<br>Multidimensional Dyspnoea Profile (MDP)<br>Medical Research Council (MRC) dyspnoea scale<br>Baseline Dyspnoea Index (BDI) and Transition Dyspnoea Index (TDI)
Physical Activity	Activity monitors (GENEActiv and ActiGraph devices) to measure daily step count, sedentary time, moderate and vigorous activity
Exercise capacity	Incremental Shuttle Walk Test (ISWT)
Frailty	Short Performance Physical Battery (SPPB), Fried's frailty score, Rockwood frailty score, handgrip and quadriceps strength.

For peer review only - http://bmjopen.bmj.com/site/about/guidelines.xhtml

Methods and Analysis

This is a mixed methods study designed using the Medical Research Council Guidelines on developing complex interventions (23).

Trial Design and Registration

This is a one-year feasibility cluster randomised controlled trial recruiting from primary care. Ten GP practices from East and West Leicestershire and Leicester City clinical commissioning groups (CCG) will be cluster randomised to a structured diagnostic pathway or usual care. The intervention practices will follow a structured diagnostic pathway to include early investigations. Usual care will continue without any intervention.

The University of Leicester will act as study sponsor and the trial has been registered on the ISRCTN website (ISRCTN14483247).

Patient and Public Involvement

Prior to the trial design an engagement event was held with clinicians and patients from relevant services, including GPs, community and hospital clinicians with cardiorespiratory background, and patients with experience of chronic breathlessness to discuss the optimal breathlessness pathway using Listening into Action (24). The structured pathway to be used in the trial was the output from this engagement, the NICE guidance and the Breathlessness IMPRESS Tips for Clinicians guidance (12, 14).

The NIHR Biomedical Research Centre Patient and Public Involvement (PPI) groups were also consulted about the study design including the duration of the research visits, patient facing information and questionnaire packs. They provided feedback on the type and ordering of the

For peer review only - http://bmjopen.bmj.com/site/about/guidelines.xhtml

questionnaires and ways to reduce burden to patients. The wording for the electronic template to aid recruitment was developed by members of the PPI group. The trial management and steering groups will have patient members. The study team aim to feed back to all local PPI partners with results from this trial.

Participants

Eligibility Criteria for Patients

Patients will be eligible if they are over 40 years old, experienced breathlessness for over two months, and are within their first two presentations to primary care with symptoms of breathlessness. Exclusion criteria are an existing diagnosis for their current symptom of breathlessness, an estimated prognosis of less than one year, or if the patient requires immediate hospitalisation for their symptoms.

Eligibility Criteria for GP practices

GP practices will be approached to take part in this study if they serve a patient population over ten thousand. Practices that are research active, as identified by the local Clinical Research Network (CRN) research scheme, will be approached. The practices will be visited by the study team to discuss taking part in the study and engage with the practice teams.

The GP practices will be randomised 1:1 and stratified by CCG using Statarand (25), a Stata randomisation module (Boston College Department of Economics).

Recruitment

Patients will be recruited over one year. Patients who meet the above eligibility criteria will be approached in primary care when they present with symptoms of chronic breathlessness. An

For peer review only - http://bmjopen.bmj.com/site/about/guidelines.xhtml

electronic template on the patient record, triggered at the point of consultation, will be used to aid opportunistic recruitment (see Figure 1). The template will be triggered by either free text or Read codes relating to breathlessness. Limits have been set on the trigger to avoid it appearing for patients who have an existing diagnosis of COPD or HF. The template summarises the study, prompting the GP to ask if the patient gives consent to be contacted by the study team. The GP will select yes or no as appropriate to the patient agreeing to have their contact details sent to the study team.

The electronic template has been developed in partnership with Keele Clinical Trials Unit who will support implementation onto the electronic patient record system (SystmOne and EMIS) for each practice. This approach has been used successfully in other primary care trials (26). The Leicester City Clinical Commissioning Group (CCG) who take the research lead for Leicester and Leicestershire were consulted regarding the use of the electronic template.

[Figure 1.]

Setting

The GP practices are located in Leicester and Leicestershire, England, UK. The research team is based at Glenfield hospital, University Hospitals of Leicester NHS Trust. Participants will be invited to attend Glenfield hospital for a research visit.

Safety Reporting

Participation is considered to be low risk. It is believed the occurrence of any serious adverse events (SAEs) will be low. Participants will be undertaking some physical tests as part of the research. There may be a small risk of worsening breathlessness, changes in blood pressure and changes in heart rate

For peer review only - http://bmjopen.bmj.com/site/about/guidelines.xhtml

and a very small risk of falls. Trained staff and emergency equipment will be available to deal with any serious events. All adverse events and serious adverse events will be recorded on the adverse event log. Any SAEs related to the physical tests conducted as part of the study will be reported.

Procedure

A weekly search will be performed by practice staff to provide a report of patients who have agreed to be contacted by the study team following discussion with the GP, as described above. The report will be sent via secure encrypted nhs.net email and patients will be contacted to complete telephone screening for eligibility. A script for telephone screening will be available for use to confirm patients' age, previous medical history and to explain the study in more detail. If patients agree to hear more about the study they will be sent a participant information sheet by post and, where willing, a provisional appointment letter. When patients attend their research visit, they will complete written informed consent (online supplement file 1) and their GP will be notified of their involvement in the study. For patients who decline to take part in the study or are ineligible, the reasons will be documented and collated.

Where possible, patients will attend their research visit within one month of seeing the GP with their breathlessness symptoms. Patients will attend a second research visit 12 months after their initial appointment and also be contacted by phone at six months and asked to complete the questionnaire pack, which will be sent in the post. Please see figure 2. for the study schedule.

[Figure 2. Study Schedule]

For peer review only - http://bmjopen.bmj.com/site/about/guidelines.xhtml

Intervention – structured diagnostic pathway

Patients who attend GP surgeries in the intervention group will undergo a set of investigations within one month; body mass index (BMI), spirometry, electrocardiogram (ECG), chest X-ray (CXR), Full blood count (FBC), N-terminal (NT)-pro hormone BNP (NT-proBNP) profile, anxiety and depression screening using the Patient Health Questionnaire – 4 item (PHQ-4) (27) and the General Practice Physical Activity Questionnaire (GPPAQ) (28). The GPs and clinicians will be provided with the pathway document to support a structured history, examination and use of the investigations (Figure 3). The electronic template will prompt the GP to action these investigations. The pathway will also be provided as a laminated document for each clinician in the Intervention practices, with small laminated flash cards of the investigations available on the work station. The pathway will recommend that patients are reviewed, along with their results, within one month and appropriate next steps to be taken regarding patient management.

Usual care will also have the electronic template triggered but will only ask the patient for their consent to pass on contact details to the study team. The GPs and clinicians in usual care will be asked to proceed with investigating the patient and their symptoms as per their usual practice and be directed to the NICE Clinical Knowledge summary for Breathlessness(14) to standardise care.

[Figure 3. Diagnostic Pathway]

Outcomes

As this is a feasibility trial, a formal sample size/power calculation is not required. Recruitment itself is one of the main measures of feasibility. The feasibility measures are outlined in Table 1 and the secondary outcome measures in Table 2. Recruitment rate will be recorded as a proportion of

For peer review only - http://bmjopen.bmj.com/site/about/guidelines.xhtml

participants consented compared to the number of participants identified as eligible by the GP practices.

Data Collection

Data will be collected in accordance with Sponsor policies and standard operating procedures. Baseline data will be collected at research Visit 1, within one month of the patient consulting their GP for breathlessness. Questionnaires will be completed by post at six months and Visit 2 will occur at twelve months after Visit 1. Healthcare records will be reviewed for all patients at three and 12 months to record the investigations performed and when, diagnosis, and time to diagnosis.

Detailed health-care utilisation data will be recorded at 12 months and five years, including hospital admissions, healthcare use, and patient survival. This information will be collected from GP records and NHS digital.

Anthropometric Measures

Body Mass Index (BMI) will be calculated by measuring the patient's height and weight. Body composition using bioelectrical impedance will provide measurements of lean mass and body fat percentage. Each participant's waist and hip circumference will be measure to the nearest 0.1cm.

Patient reported outcome measures

Health-related quality of life

The Chronic Heart Questionnaire Self-Report (CHQ-SR) is a validated and responsive questionnaire developed for patients with heart disease to assess health-related quality of life (29). It has four domains: dyspnoea, fatigue, mastery and emotional function and a known minimal clinically important difference (MCID) in patients with chronic heart and lung disease (Chronic Respiratory

For peer review only - http://bmjopen.bmj.com/site/about/guidelines.xhtml

Questionnaire version). We will be using it in a population with undifferentiated breathlessness as part of feasibility.

The Euroqol Five Dimension Five Level questionnaire (EQ5D-5L) (30) will be used to assess generic health-related quality of life. The EQ5D-5L was chosen as it is a standardised measure of health status independent of disease used to calculate quality of life year (QALY).

Breathlessness

The following questionnaires will be used to assess different aspects of breathlessness and to help select appropriate patient reported outcome measures for the future trial:

1. Dyspnoea-12 is a brief 12 item, self-complete, questionnaire which has been found to reliably measure breathlessness in a variety of diseases [23]. Dyspnoea has both sensory and afferent components and this tool was developed to ensure both aspects could be measured.

2. The Multi-Dimensional Dyspnoea Profile (MDP) is a self-complete questionnaire for breathlessness divided into an immediate perception domain and an emotional response domain (31). This questionnaire shows responsiveness to change in an acute and routine care setting for patients with breathlessness (31).

3. The Baseline Dyspnoea Index and Transition Dyspnoea Index (BDI/TDI) are short interviewer led questionnaires involving open questions about how their breathlessness affects everyday life (32). This is measured over time in respect to what tasks a patient can manage and how much effort is required to complete a task.

4. The MRC Dyspnoea Grade five point scale is patient completed and requires participants to indicate to what extent their breathlessness limits their function by working down the statements which increase in severity regarding functional limitation. (33).

For peer review only - http://bmjopen.bmj.com/site/about/guidelines.xhtml

Anxiety and depression

Participants will also complete the Hospital Anxiety and Depression (HADS) scale which is a simple self-completed questionnaire with 14 questions relating to either anxiety or depression (34)

Activation measure

The Patient Activation Measure (PAM) is a self-completed questionnaire which assesses patients' knowledge, skills and confidence to manage their own health (35).

Physical Activity

Daily physical activity and stationary time will be assessed for seven days using the GT3x ActiGraph device that is worn around the waist (36), and the wrist worn GENEActiv device (37). Sedentary time, daily step count and time spent in moderate and vigorous physical activity will be recorded. The activity monitors will be fitted at visits 1 and 2 and will be programmed to begin collecting data at midnight. Data will then be collected for seven days thereafter. To maximise the use of the data for comparison with other disease datasets we will use both devices as long as patients are willing. If participants can only use one device we will request that this is the wrist worn GENEActiv.

Exercise capacity

This will be assessed using the Incremental Shuttle Walk Test (ISWT) (38). This walking test requires the patient to walk between two cones nine metres apart in time to a set of auditory beeps. Initially, the walking speed is very slow, but each minute the required walking speed progressively increases. The patient will walk for as long as they can until they are either too breathless or can no longer keep up with the beeps at which time the test ends. It is reported as the distance walked. The ISWT is found to be valid, reliable and responsive in patients with chronic heart and respiratory disease (39-41).

For peer review only

For peer review only - http://bmjopen.bmj.com/site/about/guidelines.xhtml

Heart rate, oxygen saturations, blood pressure and BORG breathlessness score will be recorded before and after completion of the walk tests. A practice walk test will be performed as per the guidelines for this exercise test.

Frailty

Fried's frailty definition will be recorded, which is based on patient reported weight loss and exhaustion along with measured slowness (gait speed), weakness (hand grip) and physical activity (42). This has been shown to provide a standardised definition of frailty with predictive validity in the community dwelling older population (43). The Rockwood Frailty Scale will also be recorded. This is completed by the researcher in response to medical history and outcome measures taken during the visit and has also been demonstrated as a valid and reliable way of documenting frailty (44).

Participants will also complete the Timed Up and Go test where the patient starts in a seated position, stands and walks 3 metres, then turns around and returns to the seated position (45). The patient is timed how long this process takes.

The Short Physical Performance Battery (46) which includes the Four Metre Gait Speed (4MGS) test and the Sit-to-Stand test and assessing balance with the patient standing in different positions (side by side stand, semi tandem stand, tandem stand) will be completed, along with handgrip and quadriceps strength.

Health Economic Modelling

The feasibility study is structured to support the future RCT which will estimate the lifetime incremental cost per QALY gained. The objectives are to identify: the main NHS and prescribed specialist services (PSS) cost components; the resource use and unit cost data required for each of

For peer review only - http://bmjopen.bmj.com/site/about/guidelines.xhtml

these components; potential sources of HRQOL data suitable for estimating QALYs in this patient group; potential sources that could be used to estimate residual life expectancy and other long-term outcomes among patients.

Semi-structured interviews.

Audio-recorded interviews will be conducted privately face-to-face or via telephone between the participant and an interviewer, following informed consent. Interviews will take place with patients and GPs until data saturation is perceived. The interviews are anticipated to be between 30 minutes and one hour duration and will be professionally transcribed verbatim, with identifiable information removed. The transcription will be performed by an external company and a confidentiality agreement will be in place. Interview prompts will be devised based upon relevant literature, experience of the team and consultation with patient representatives.

Patients consented for the feasibility trial who are willing and able, and healthcare staff from the participating practices, will be interviewed.

The interviews will explore patients' experience of breathlessness, taking part in the trial, and their related healthcare. Patients will also be asked about the acceptability of the research visits and outcome measures performed and about their understanding of the trial. Interviews with healthcare staff will seek to understand what is usual care and any influence that taking part in the trial has on usual care. The clinician interviews will include questions about what a diagnostic pathway should or could look like from the perspective of the health professionals. They will also explore any barriers to screening patients for eligibility, challenges in implementing the pathway, or perceived benefits of the intervention. The patient participant and clinician interview guides can be viewed in the online supplement.

For peer review only

Data Management

Paper based anonymised study records will be stored in locked filing cabinets within a locked office at Glenfield Hospital. Electronic records will be stored on a restricted access, secure University of Leicester and University Hospitals of Leicester NHS Trust computer system, maintained by the Trust. Audio recordings will be done using an encrypted Dictaphone. The recordings will be uploaded to secure files on University of Leicester and University Hospitals of Leicester computers then deleted from the Dictaphone. Access to the files will be restricted and password protected.

Data Analysis

Data analysis will be performed in an exploratory fashion. Descriptive statistics, number and percentage for categorical data and mean and standard deviation or median and inter-quartile range for non-normally distributed continuous data will be present for all demographics, baseline characteristics and questionnaire scores. Normality of the baseline characteristics will be determined using the Kolmogorov-Smirnov test (KS-test) or Shapiro-Wilkes test depending on final recruitment numbers.

SPSS version 26 will be used for statistical analysis. Graphpad software will be used for any figures. Data analysis will be performed on the complete dataset utilising all participants.

Secondary outcomes for both groups will be described as mean (SD) and median [IQR] for normally or non-normally distributed data respectively. The time to diagnosis will be analysed using survival analyses based on Cox proportional hazards survival modelling. The proportion of patients with valid diagnosis at three months and one year will be described and compared using chi squared tests.

For peer review only

For peer review only - http://bmjopen.bmj.com/site/about/guidelines.xhtml

Qualitative Analysis

The interviews will be reviewed using thematic analysis (47), supported by NVivo software. This approach follows six distinct stages: familiarisation with data; generating initial codes; searching for themes; reviewing themes; defining and naming themes and producing the report (48). Initial coding will be carried out and a sample of interviews will be coded by a second member of the team to ensure consistency and to enhance interpretive authenticity. Throughout the data analysis, an iterative approach will be undertaken with the research team meeting to discuss and review emerging themes and search for accounts that provide contesting views of the same phenomena or identify different phenomena. Analysis will continue until data saturation and themes will be synthesised and supported by using relevant quotes from the data. Patient representatives will be invited to comment on the emerging themes from the patient interviews to assess whether important issues may have been missed which could be included in subsequent interviews.

Protocol amendments

Any changes to the study protocol outlined in this paper will be approved by Nottingham 1 Research Ethics Committee. This will be in agreement with Sponsor University of Leicester and University Hospitals of Leicester Research department.

Ethics and Dissemination

Ethical Approval: The Research Ethics Committee Nottingham 1 have provided ethical approval for this research study (REC Reference: 19/EM/0201).

For peer review only - http://bmjopen.bmj.com/site/about/guidelines.xhtml

Monitoring

A Trial Steering Committee (TSC) will be convened to provide oversight and support to the project. The committee will comprise of an independent Chair, independent members including clinicians, experts in breathlessness, statistician and policy experts, patient representative members and the Principal Investigator (PI). The Trial Co-ordinator and will attend meetings as appropriate. A TSC Charter will be put in place and 'Conflict of Interest' declarations obtained for all members and attendees. The TSC will meet as required to monitor the progress of the study, adherence to the protocol, progress of the study, consideration of new information of relevance to the research question and participant safety.

A Trial Management Group (TMG) has been established during the preparation of the study. Group members include the PI, Research Associate/Project lead, Trial Co-ordinator and Research Assistants. Other collaborators and Leicester Clinical Trials Unit (LCTU), specialities with specific expertise will attend as appropriate. The TMG will be held at least monthly to monitor all aspects of the conduct and progress of the study, ensure that the protocol is adhered to and take appropriate action to safeguard participants and the quality of the study itself.

Dissemination

Results from the study will be disseminated by presentations at relevant meetings and conferences including British Thoracic Society (BTS) and Primary Care Respiratory Society (PCRS), as well as by peer-reviewed publications and through patient presentations and newsletters to patients, where

For peer review only - http://bmjopen.bmj.com/site/about/guidelines.xhtml

available. The results will also be shared with local primary and secondary care partners. Following the feasibility trial, the aim is to conduct a national multicentre trial to assess clinical and cost effectiveness of a diagnostic pathway for breathlessness. The feasibility outcomes collected and qualitative analysis will help refine the design of a future trial.

Funding statement: This work was funded by a NIHR Clinician Scientist Fellowship (CS-2016-16-020) awarded to Dr Rachael A Evans. Professor Natalie Armstrong is supported by a Health Foundation Improvement Science Fellowship and also by the National Institute for Health Research (NIHR) Applied Research Collaboration East Midlands (ARC EM). The views expressed are those of the authors and not necessarily those of the National Health Service, the NIHR, or the Department of Health.

Competing interests: The authors declare no competing interests.

Authors' contributions: RAE conceived the research idea, and developed the theory and plan for this study. GD and RAE drafted the initial manuscript. SW developed the Electronic template for use in the recruitment strategy. All authors (RAE, GD, JC, SW, SC, SE, NB, DJ, NA and MS) contributed to the study development and reviewed, commented and approved the manuscript.

Acknowledgements: We acknowledge and are grateful to our public and patient involvement (PPI) members; Winifred Smart, Jagruti Lalseta, Brian Davies, Paul Ashby and Tony Watling. RAE thanks Professor Mike Morgan and the Research Design Service, University of Leicester for their support with the NIHR clinician scientist fellowship application. We also thank the clinicians who contributed to the development of the structured diagnostic pathway including Professor Ruth

For peer review only - http://bmjopen.bmj.com/site/about/guidelines.xhtml

Green, Mrs Louise Clayton, Mrs Karen Moore, Mr Alex Woodward, Mrs Jo Szymkowiak, Ms Alison Scott, Ms Jane Giles. The work is supported by the NIHR Leicester Biomedical Research Centre-Respiratory.

For peer review only

For peer review only - http://bmjopen.bmj.com/site/about/guidelines.xhtml

References

1. Hutchinson A, Pickering A, Williams P et al. Breathlessness and presentation to the emergency department: a survey and clinical record review. *BMC Pulm Med* 2017;17(1):53.
2. Laribi S, Keijzers G, van Meer O, et al. Epidemiology of patients presenting with dyspnea to emergency departments in Europe and the Asia-Pacific region. *European Journal of Emergency Medicine*2019;26(5):345-9.
3. Frese T, Sobeck C, Herrmann K et al. Dyspnea as the reason for encounter in general practice. *J Clin Med Res* 2011;3(5):239-46.
4. Stevens JP, Dechen T, Schwartzstein R et al. Prevalence of Dyspnea Among Hospitalized Patients at the Time of Admission. *Journal of pain and symptom management* 2018;56(1):15-22.e2.
5. Currow DC, Plummer JL, Crockett A et al.. A community population survey of prevalence and severity of dyspnea in adults. *J Pain Symptom Manage* 2009;38(4):533-45.
6. Bowden JA, To TH, Abernethy AP et al. Predictors of chronic breathlessness: a large population study. *BMC Public Health* 2011;11:33.
7. Gronseth R, Vollmer WM, Hardie JA et al. Predictors of dyspnoea prevalence: results from the BOLD study. *Eur Respir J* 2014;43(6):1610-20.
8. van Mourik Y, Rutten FH, Moons KG et al. Prevalence and underlying causes of dyspnoea in older people: a systematic review. *Age Ageing* 2014;43(3):319-26.
9. Smith AK, Currow DC, Abernethy APet al. Prevalence and Outcomes of Breathlessness in Older Adults: A National Population Study. *Journal of the American Geriatrics Society* 2016;64(10):2035-41.
10. Frostad A, Soyseth V, Andersen A et al. Respiratory symptoms as predictors of all-cause mortality in an urban community: a 30-year follow-up. *J Intern Med* 2006;259(5):520-9.
11. Gillespie DJ, Staats BA. Unexplained dyspnea. *Mayo Clin Proc* 1994;69(7):657-63.
12. IMPRESS. Breathlessness IMPRESS Tips (BITs) For clinicians 2016 [Available from: https://www.respiratoryfutures.org.uk/resources/impress-documents/impress-breathlessness-algorithm/.
13. Sandberg J, Ekstrom M, Borjesson M et al. Underlying contributing conditions to breathlessness among middle-aged individuals in the general population: a cross-sectional study. *BMJ Open Respir Res* 2020;7(1).
14. NICE. Clinical Knowledge Summary on Breathlessness 2017 [Available from: https://cks.nice.org.uk/breathlessness.
15. Jones RC, Price D, Ryan D et al. Opportunities to diagnose chronic obstructive pulmonary disease in routine care in the UK: a retrospective study of a clinical cohort. *Lancet Respir Med* 2014;2(4):267-76.
16. Bottle A, Kim D, Aylin P et al. Routes to diagnosis of heart failure: observational study using linked data in England. *Heart* 2018;104(7):600-5.
17. Collard HR, Tino G, Noble PW et al. Patient experiences with pulmonary fibrosis. *Respir Med* 2007;101(6):1350-4.
18. du Bois RM. An earlier and more confident diagnosis of idiopathic pulmonary fibrosis. *Eur Respir Rev*2012;21(124):141-6.
19. Aaron SD, Boulet LP, Reddel HK et al. Underdiagnosis and Overdiagnosis of Asthma. *American Journal of Respiratory and Critical Care Medicine* 2018;198(8):1012-20.
20. Heffler E, Crimi C, Mancuso S et al. Misdiagnosis of asthma and COPD and underuse of spirometry in primary care unselected patients. *Respir Med* 2018;142:48-52.
21. Royal College of Physicians. National Asthma and COPD Audit Programme (NACAP): primary care workstream. Wales primary care clinical audit report 2020.: RCP; 2020.
22. Singh H, Dickinson JA, Theriault G et al. Overdiagnosis: causes and consequences in primary health care. *Can Fam Physician* 2018;64(9):654-9.

For peer review only - http://bmjopen.bmj.com/site/about/guidelines.xhtml

23. Medical Research Council. Developing and evaluating complex interventions. 2019.
24. Listening into Action 2019 [Available from: http://www.listeningintoaction.co.uk/LiA-info/index.php.
25. Ternovski J. STRATARAND: Stata module to perform stratified random assignment. 2015.
26. Foster N, Mullis R, Hill J et al. Effect of Stratified Care for Low Back Pain in Family Practice (IMPaCT Back): A Prospective Population-Based Sequential Comparison. *The Annals of Family Medicine* 2014;12:102-11.
27. Kroenke K, Spitzer RL, Williams JB et al. An ultra-brief screening scale for anxiety and depression: the PHQ-4. *Psychosomatics* 2009;50(6):613-21.
28. Department of Health and Social Care. General Practice Physical Activity Questionnaire. 2013.
29. Evans RA, Singh SJ, Williams JE et al. The development of a self-reported version of the chronic heart questionnaire. *J Cardiopulm Rehabil Prev* 2011;31(6):365-72.
30. (5Q5D5L). E. Euroqol (5Q5D-5L) 2016 [Available from: www.euroqol.org/eq-5d-products/eq-5d-5l.html .
31. Meek PM, Banzett R, Parsall MB et al. Reliability and Validity of the Multidimensional Dyspnea Profile. *Chest* 2012;141(6):1546-53.
32. Witek TJ, Jr., Mahler DA. Minimal important difference of the transition dyspnoea index in a multinational clinical trial. *Eur Respir J* 2003;21(2):267-72.
33. Fletcher CM, Elmes PC, Fairbairn AS et al. The significance of respiratory symptoms and the diagnosis of chronic bronchitis in a working population. *Br Med J* 1959;2(5147):257-66.
34. Zigmond AS, Snaith RP. The hospital anxiety and depression scale. *Acta Psychiatr Scand* 1983;67(6):361-70.
35. Hibbard JH, Mahoney ER, Stockard J et al. Development and testing of a short form of the patient activation measure. *Health Serv Res* 2005;40(6 Pt 1):1918-30.
36. Actigraph. [Available from: https://actigraphcorp.com/support/activity-monitors/gt3x/
37. GeneActiv. [Available from: https://www.activinsights.com/actigraphy/geneactiv-original/
38. Singh SJ, Morgan MD, Scott S et al. Development of a shuttle walking test of disability in patients with chronic airways obstruction. *Thorax* 1992;47(12):1019-24.
39. Singh SJ, Puhan MA, Andrianopoulos V et al. An official systematic review of the European Respiratory Society/American Thoracic Society: measurement properties of field walking tests in chronic respiratory disease. *Eur Respir J* 2014;44(6):1447-78.
40. Holland AE, Spruit MA, Troosters T et al. An official European Respiratory Society/American Thoracic Society technical standard: field walking tests in chronic respiratory disease. *Eur Respir J* 2014;44(6):1428-46.
41. Morales FJ, Martínez A, Méndez M, et al. A shuttle walk test for assessment of functional capacity in chronic heart failure. *Am Heart J* 1999;138(2 Pt 1):291-8.
42. Fried LP, Tangen CM, Walston J et al. Frailty in older adults: evidence for a phenotype. *J Gerontol A Biol Sci Med Sci* 2001;56(3):M146-56.
43. Newman AB, Hirsch C, Tangen CM et al. Frailty in Older Adults: Evidence for a Phenotype. *The Journals of Gerontology: Series A* 2001;56(3):M146-M57.
44. Rockwood K, Song X, MacKnight C et al. A global clinical measure of fitness and frailty in elderly people. *CMAJ* 2005;173(5):489-95.
45. Podsiadlo D, Richardson S. The timed "Up & Go": a test of basic functional mobility for frail elderly persons. *J Am Geriatr Soc*1991;39(2):142-8.
46. Guralnik JM, Simonsick EM, Ferrucci L et al. A short physical performance battery assessing lower extremity function: association with self-reported disability and prediction of mortality and nursing home admission. *J Gerontol* 1994;49(2):M85-94.
47. Braun V, Clarke V. Reflecting on reflexive thematic analysis. *Qual Res Sport Exerc* 2019;11(4):589-97.
48. Clarke V, Braun V. Thematic analysis. *J Posit Psychol* 2017;12(3):297-8.

For peer review only - http://bmjopen.bmj.com/site/about/guidelines.xhtml

Figure 1. The electronic template triggered on patient electronic healthcare record

Figure 2. Study Schedule

Figure 3. Diagnostic Pathway

For peer review only

For peer review only - http://bmjopen.bmj.com/site/about/guidelines.xhtml

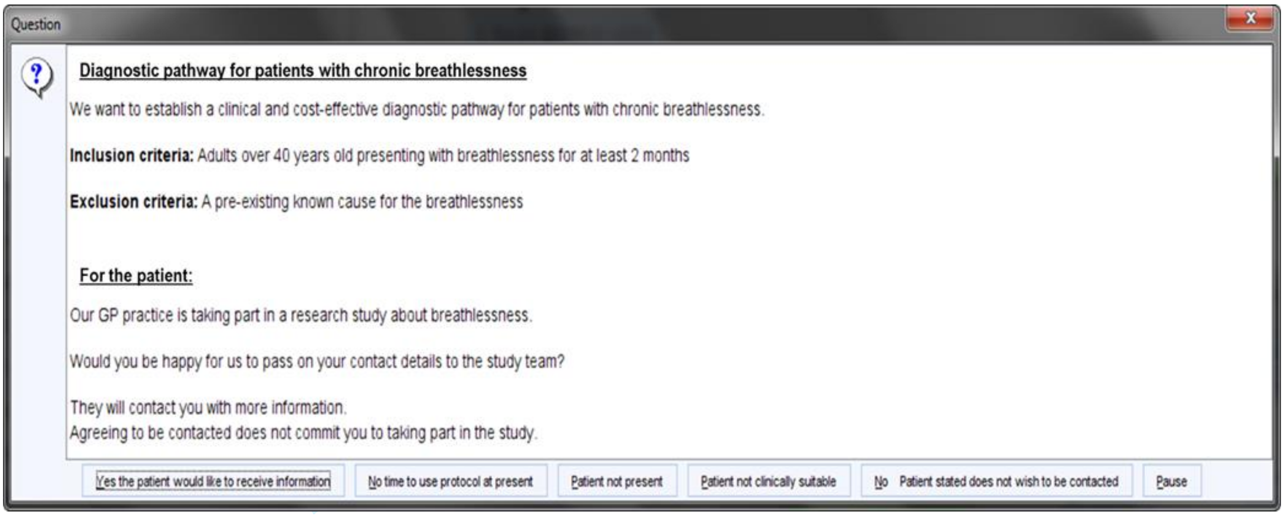

Question

Diagnostic pathway for patients with chronic breathlessness

We want to establish a clinical and cost-effective diagnostic pathway for patients with chronic breathlessness.

Inclusion criteria: Adults over 40 years old presenting with breathlessness for at least 2 months

Exclusion criteria: A pre-existing known cause for the breathlessness

For the patient:

Our GP practice is taking part in a research study about breathlessness.

Would you be happy for us to pass on your contact details to the study team?

They will contact you with more information.
Agreeing to be contacted does not commit you to taking part in the study.

Yes the patient would like to receive information | No time to use protocol at present | Patient not present | Patient not clinically suitable | No Patient stated does not wish to be contacted | Pause

For peer review only

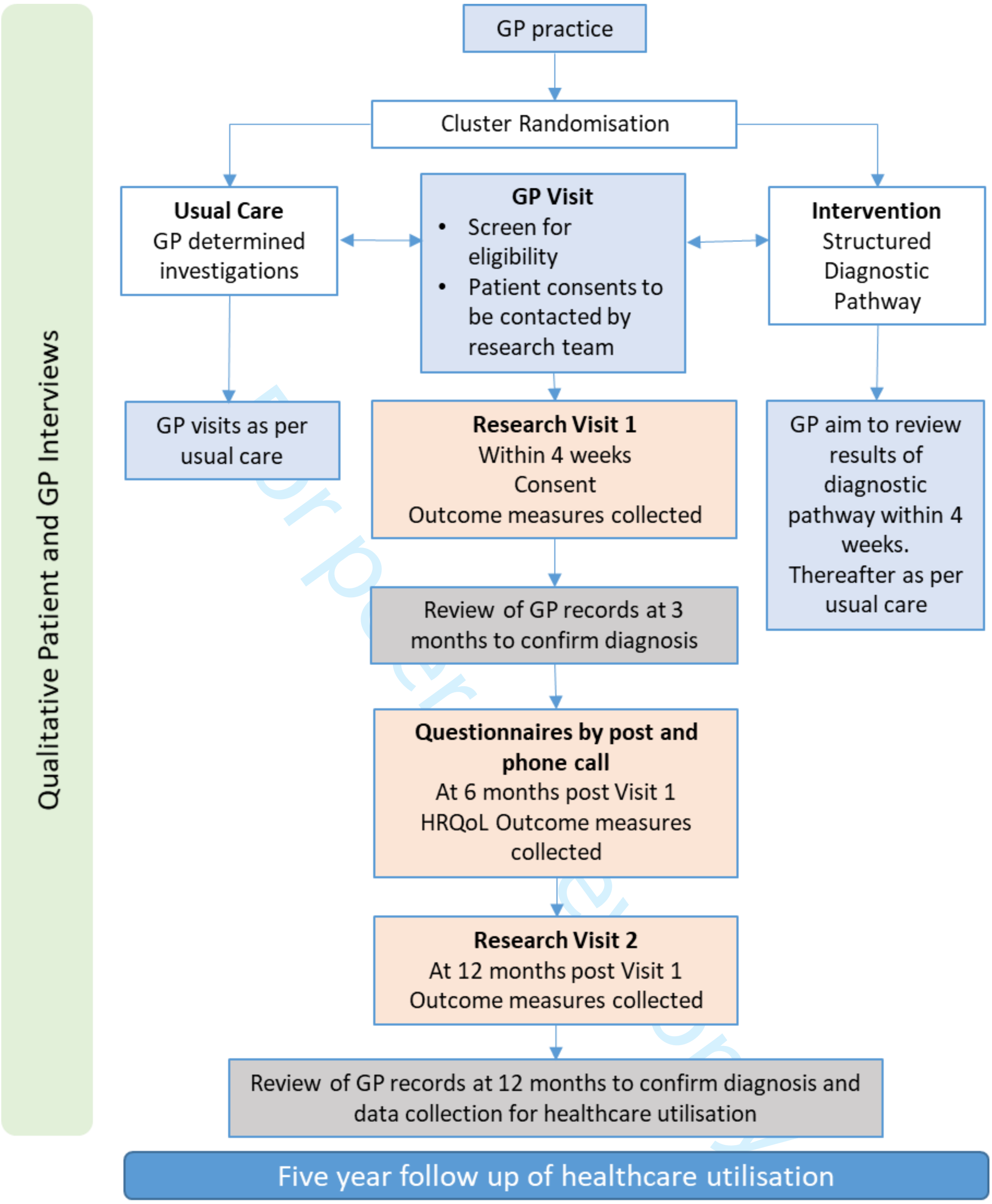
Qualitative Patient and GP Interviews
GP practice
Cluster Randomisation
Usual Care
GP determined investigations
GP Visit
Screen for eligibility
Patient consents to be contacted by research team
Intervention
Structured Diagnostic Pathway
GP visits as per usual care
Research Visit 1
Within 4 weeks
Consent
Outcome measures collected
GP aim to review results of diagnostic pathway within 4 weeks.
Thereafter as per usual care
Review of GP records at 3 months to confirm diagnosis
Questionnaires by post and phone call
At 6 months post Visit 1
HRQoL Outcome measures collected
Research Visit 2
At 12 months post Visit 1
Outcome measures collected
Review of GP records at 12 months to confirm diagnosis and data collection for healthcare utilisation
Five year follow up of healthcare utilisation

NIHR National Institute for Health Research (Funded by) · University of Leicester · NHS University Hospitals of Leicester NHS Trust · Breathlessness Diagnostic Pathway

Diagnostic pathway for initial presentation of chronic breathlessness

For adults > 40 yrs old presenting with breathlessness for > 2 months please follow this pathway.

Please Read code for Breathlessness. For acute breathlessness, please follow usual procedure for assessment and action.

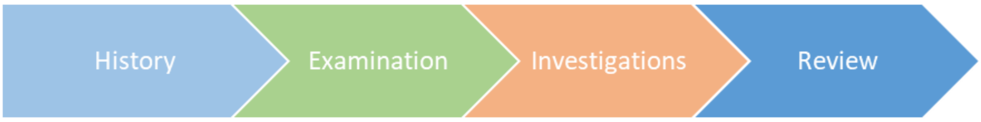

STEP 1. Focused History and Examination

As usual, please take a clear history to assess possible causes and impact of breathlessness. Please include the following:

HISTORY

- ✓ Onset and duration of breathlessness
- ✓ At rest/exertional
- ✓ Nature of breathlessness
- ✓ Aggravating and relieving factors
- ✓ Associated symptoms (e.g. chest pain, cough, wheeze, ankle swelling, palpitations)
- ✓ Orthopnoea, PND
- ✓ Levels of exercise and daily activity
- ✓ Impact on everyday life and MRC Dyspnoea scale
- ✓ Co-morbidities and medications
- ✓ Smoking history including pack years and substance smoked
- ✓ Environmental and occupational risk factors

EXAMINATION

- ✓ Vital signs: resting HR and rhythm, O_2 saturation, RR and BP
- ✓ Observe general appearance and breathing pattern (increase use of accessory muscles)
- ✓ Assess JVP
- ✓ Check for peripheral oedema
- ✓ Auscultate lungs (particularly for bi-basal crackles)
- ✓ Auscultate cardiac sounds (listen for murmurs including right carotid area for aortic stenosis)
- ✓ BMI (weight kg/height m^2)

For peer review only

STEP 2. Investigations for Chronic Breathlessness

Please initiate the investigations listed below to rule in/out common causes of breathlessness.

Investigation	Implications for diagnosis
**Body Mass Index (BMI)**	To diagnose obesity if >30kg/m^2
**ECG**	To detect arrhythmias or diagnoses suggestive of ischaemic heart disease
**Chest X-ray**	To detect signs of heart failure or pulmonary pathology e.g. pleural effusion, interstitial changes
**Spirometry** (If FEV_1/FEV_6 <0.75 perform full spirometry)	Obstructive pattern (FEV_1/FVC < 0.70) may suggest airway disease such as COPD or asthma Restrictive pattern (FEV_1/FVC > 0.70 but FEV_1 and FVC < 80% predicted) may suggest conditions such as obesity, interstitial lung disease, chest wall abnormality or neuromuscular disease
**FBC**	Haemoglobin to exclude anaemia Eosinophil count if >0.3 x10^9/L can support a diagnosis of asthma
**BNP profile**	To exclude heart failure
**Urea and electrolytes/ TSH**	To exclude metabolic causes
**Activity Questionnaire GPPAQ**	To assess physical activity levels
**PHQ4**	Screening questionnaires for symptoms of anxiety and depression

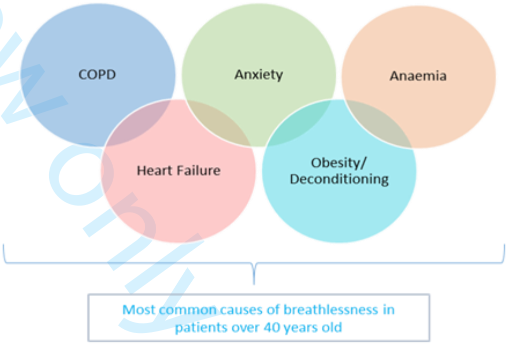

MRC Grade	Degree of breathlessness related to activity
1	Not troubled by breathlessness except on strenuous exercise
2	Short of breath when hurrying on level ground or when walking up a slight hill
3	Walks slower than contemporaries on level ground due to breathlessness, or has to stop for breath when walking at own pace
4	Stops for breath after walking about 100m or after a few minutes on level ground
5	Too breathless to leave the house, or breathless when dressing or undressing

STEP 3. Please aim to review results and patient within 1 month. Please use additional document for guidelines and onward referral as appropriate

For peer review only - http://bmjopen.bmj.com/site/about/guidelines.xhtml

Diagnostic Uncertainty → Please consider referral to Breathlessness Clinic (referral form in PRISM)

FUNDED BY NIHR | National Institute for Health Research
UNIVERSITY OF LEICESTER
NHS University Hospitals of Leicester NHS Trust
Breathlessness Diagnostic Pathway

CONSENT FORM

Breathlessness Study – Diagnostic Pathway

Site: Glenfield Hospital	Participant ID No:
Sponsor: University of Leicester	Name of researcher:

Please initial in the box

1. I confirm that I have read and understand the Participant Information Sheet **version no. ___dated ____**for the above study, and have had the opportunity to ask questions. ☐
2. I understand that my participation is voluntary and that I am free to withdraw at any time without giving a reason, and without my medical care or legal rights being affected. I understand that any data collected up until my withdrawal will still be used in the research study. ☐
3. I agree to undergo the tests and investigations described in the Participant Information Sheet. The nature of the tests, investigations and any possible risks have been explained. ☐
4. I understand that relevant sections of my medical records and data collected during the study may be looked at by individuals from the study team, Leicester Clinical Trials Unit, the Sponsor, regulatory authorities or the NHS Trust, where it is relevant to my taking part in this research. I give permission for these individuals to have access to my records. ☐
5. I give permission to the study team to retrieve data about the healthcare services I have utilised over the 12 months of the study. ☐
6. I understand that the information held and maintained by the Health and Social Care Information Centre (NHS Digital) and other central UK NHS bodies may be accessed by research team to obtain information about my health status at one and five years. I give permission to the study team to access the data by linking it to my identifiable information. ☐
7. I agree to my GP being informed of my participation in the study. ☐
8. I understand that my personal details and study data will be stored on secure University of Leicester and University Hospitals of Leicester systems and paper copies will be kept in a secure office environment at Glenfield Hospital. ☐
9. I agree that the information collected about me will be used to support other research in the future, and may be shared anonymously with other researchers. ☐
10. I agree that some of my anonymised data will be shared with providers of the questionnaire license holders for the purpose of improving how the questionnaires are used. ☐
11. I agree to take part in an interview to find out about my experiences of breathlessness and healthcare. I understand interviews will be recorded and transcribed. ☐
12. I agree to take part in the above study. ☐

________________	__________	______________
Name of participant (printed)	Date	Signature
________________	__________	______________
Name of person taking consent (printed)	Date	Signature

When completed: 1 for participant; 1 (original) for ISF; 1 to GP

For peer review only - http://bmjopen.bmj.com/site/about/guidelines.xhtml

Study aims

- To conduct nested qualitative interviews with patients and clinicians to understand how **acceptable the trial design** is including using the pathway, patient research visits and use of outcome measures.
- To conduct nested interviews to **understand more about patient experiences of breathlessness, the process of diagnosis and how it affects their quality of life.**
- To also understand **from clinicians** more about 'usual care' and current practices and attitudes in primary care towards breathlessness as a symptom.

Interview Schedule: Understanding patient experiences in taking part in the Breathlessness Diagnostic Pathway study

Introduce self: name and role

Withdrawal

If anything we speak about today makes you feel uncomfortable you are free to not answer a particular question, request for the recorder to be switched off to resume the interview after a short break, or you can ask to terminate the interview all together at any point.

Before we start the interview I would like to collect some information about yourself:

Background information from participant: To be recorded on CRF

- **(Covid Y/N, Covid symptoms Y/N)** -maybe leave till later unless they volunteer but ensure it is recorded by end of the interview?

Thank you. We will now proceed to the interview:

Seek consent to continue and to audio-record the interview.

Let them know that no personal identifiable data will be recorded and a participant number will be allocated to them. There are no right or wrong answers here; we are just trying to understand what things are like for you.

Opening (to understand what the patient knows about the trial and has done so far in terms of visits).

- **Can you talk me through why you're here?/ What you think the study is about?/How you started on the study?**
    - What have you done so far? How has that been?

For peer review only - http://bmjopen.bmj.com/site/about/guidelines.xhtml

1: Experiences of breathlessness

- Can you tell me about your breathlessness?

  [**prompt** – I really want to understand what its like for you…. Can you tell me a bit more about that…]

2: COVID-19

- **Can you tell me about your normal day at the moment?**
- **How are you managing**? (with the current situation)

  (Going out? Social shielding? Contact with others? Anxiety?)

  - Can you describe what your health is like at the moment?

  (Symptoms of covid?)

  - How are you keeping in contact with people/if anyone?
  - Does the patient mention their breathlessness with regards to government guidelines here? If not, prompt?
  - If not reported prompts: coping, exercise, social life, worrying, depressed, isolated, existential crisis/future worries, and if seeking help – how?

- Can you tell me about any changes to your health care in the current situation (COVID-19)?

  (e.g. prescriptions, routine appointments)

Possible prompt (How is the current pandemic affecting you?

3: Experiences of breathlessness

- Thinking back, leading up to you going to the GP, can you talk me through how breathlessness was affecting you? [what led you to go to the GP?]
  - Can you tell me about when you first noticed you were becoming more breathless?
  - What made you visit your GP?
    - (Reasons, worries, what did they think might be causing it, ? prompted by family, ? stopped being able to manage certain things/jobs)
    - (Did they go for breathlessness or was it something else and GP picked up the breathlessness?)

For peer review only - http://bmjopen.bmj.com/site/about/guidelines.xhtml

- What were you expecting might happen?
- Can you tell me what think is causing your breathlessness?
  - What do you understand about that?

4: Healthcare experiences

- **Thinking about your breathlessness**, can you talk me through what's happened so far? Medical pathway / who you've been to see to, tests done, diagnosis ?
  - What do you understand about the diagnosis**?**

5: Lifestyle

- Does anything help you with your breathlessness? (are they actively coping? If so where have they found out how to do this, where have they looked for information)
  - Where did you come across this? information source /worked it out themselves?
  - Has it changed since seeking help?
  - How does it affect your everyday life?

6: Experience of taking part in the study

- In relation to this research study, what has been your experience of taking part?
  - What were the positive things?
  - What has been difficult about taking part?
  - Is there anything you think could be changed

Is there anything that we haven't covered in the interview that you think we should know or think about?

For peer review only - http://bmjopen.bmj.com/site/about/guidelines.xhtml

<u>Interview Schedule:</u> Understanding GP experiences in taking part in the Breathlessness Diagnostic Pathway study

Introduce self: name and role

Let them know that no personal identifiable data will be recorded and a participant number will be allocated to them.

Withdrawal

If anything we speak about today makes you feel uncomfortable you are free to not answer a particular question, request for the recorder to be switched off to resume the interview after a short break, or you can ask to terminate the interview all together at any point.

Before we start the interview I would like to collect some information about yourself: (Background information from participant: To be recorded)

Thank you. We will now proceed to the interview: *Seek consent to continue and to audio-record the interview.*

<u>General/context</u>

- Can you tell me a bit about how you manage breathlessness?
  - What difficulties/problems/find hard about breathlessness?
    OR
  - Is there anything you find difficult about breathlessness?
- Tell me about the last person you managed with breathlessness OR Can you think of a challenging experience with a patient with breathlessness? OR Can you tell me an instance where arriving at a diagnosis for a patient with chronic breathlessness has been difficult?
  - *Why was it challenging?*
  - *Feelings, worries, support*

(Try to see if they are focusing on disease or on breathlessness management too? Note for interviewer…)

Tell me how it really works in practice (verses ideal world)

What about managing the symptoms for that patient?

Can you tell me what you do if/when you've run all your tests and you don't find a particular cause/reason for their breathlessness?

And how do you phrase that information to the patient?

- How do you make decisions about referral?
  - *How do you make a decision that this person's breathlessness merits assessment and further management?*

- Thinking about **assessment**: How do you think your colleagues feel about assessing patients with breathlessness? (Do you think your assessment is different to your colleagues?)
- Are there conditions that you or colleagues worry about missing or are harder to pick up?
  - Why is that?

- Thinking about breathlessness **management**: How do you go about managing patients with breathlessness? (Do you colleagues work in s similar/different way?)

For peer review only - http://bmjopen.bmj.com/site/about/guidelines.xhtml

- Who else might help with the patient management (in the practice, in the area, onward referral, PR)?

  (Possible prompt: what is it that makes breathlessness different as a symptom to say back pain for example in terms of how it is assessed?)

- Thinking about patients living with chronic breathlessness: in your experience are patients coming to seek help because it has got worse (urgently seeking help) or coming in with another problem? What do you think………

Thinking about the study specifically and the diagnostic pathways for breathlessness

- What are the barriers to doing investigations or making a diagnosis?
- What would be a red flag that would make certain investigations more of priority or increase the urgency of getting them done?

What do you think an effective pathway for breathlessness should look like?

- Spirometry
- Diagnostic Hubs
- Referral
- Coding for breathlessness
- *Tests eg. CXR, blood tests – are they helpful? Where does that get you?*

  What would help or influence your clinical practice with patients with breathlessness?

Covid-19 (this might get picked up earlier in discussion)

- How are you managing with routine care within the practice at present? (Workload, structural changes/processes)
- How do you think patients are managing?
- How are you finding things are going with your patients with chronic breathlessness under the present conditions?
- How do think assessment or diagnosis is affected by COVID?

Any changes in everyday practice

In relation to this research project – can you tell me about your experience of taking part in the study so far?

Can you talk me through how the pop up worked for you?

- *Intervention: Can you tell me how being in the trial has altered your practice?*
- *Usual care: Can you tell me if being in the trial has had any effect on your practice?*

About the study

- In relation to this research study, what has been your experience of taking part?
  - Is there anything you think could be changed?

Is there anything that we haven't covered in the interview that you think we should know or think about?

For peer review only - http://bmjopen.bmj.com/site/about/guidelines.xhtml
For peer review only

STANDARD PROTOCOL ITEMS: RECOMMENDATIONS FOR INTERVENTIONAL TRIALS

SPIRIT 2013 Checklist: Recommended items to address in a clinical trial protocol and related documents*

Section/item	Item No	Description	Page number and details
**Administrative information**			
Title	1	Descriptive title identifying the study design, population, interventions, and, if applicable, trial acronym	1
Trial registration	2a	Trial identifier and registry name. If not yet registered, name of intended registry	3<br>ISRCTN14483247
	2b	All items from the World Health Organization Trial Registration Data Set	
Protocol version	3	Date and version identifier	Version 1 for publication.
Funding	4	Sources and types of financial, material, and other support	20<br>Funded by a NIHR Clinician Scientist Fellowship (CS-2016-16-020) awarded to Dr Rachael A Evans.
Roles and responsibilities	5a	Names, affiliations, and roles of protocol contributors	1
	5b	Name and contact information for the trial sponsor	7
	5c	Role of study sponsor and funders, if any, in study design; collection, management, analysis, and interpretation of data; writing of the report; and the decision to submit the report for publication, including whether they will have ultimate authority over any of these activities	12 and 18
	5d	Composition, roles, and responsibilities of the coordinating centre, steering committee, endpoint adjudication committee, data management team, and other individuals or groups overseeing the trial, if applicable (see Item 21a for data monitoring committee)	18 and 19

For peer review only - http://bmjopen.bmj.com/site/about/guidelines.xhtml

**Introduction**			
Background and rationale	6a	Description of research question and justification for undertaking the trial, including summary of relevant studies (published and unpublished) examining benefits and harms for each intervention	4 and 5
	6b	Explanation for choice of comparators	
Objectives	7	Specific objectives or hypotheses	5
Trial design	8	Description of trial design including type of trial (eg, parallel group, crossover, factorial, single group), allocation ratio, and framework (eg, superiority, equivalence, noninferiority, exploratory)	7<br>Feasibility cluster randomised controlled trial.
**Methods: Participants, interventions, and outcomes**			
Study setting	9	Description of study settings (eg, community clinic, academic hospital) and list of countries where data will be collected. Reference to where list of study sites can be obtained	9
Eligibility criteria	10	Inclusion and exclusion criteria for participants. If applicable, eligibility criteria for study centres and individuals who will perform the interventions (eg, surgeons, psychotherapists)	8
Interventions	11a	Interventions for each group with sufficient detail to allow replication, including how and when they will be administered	11
	11b	Criteria for discontinuing or modifying allocated interventions for a given trial participant (eg, drug dose change in response to harms, participant request, or improving/worsening disease)	N/A
	11c	Strategies to improve adherence to intervention protocols, and any procedures for monitoring adherence (eg, drug tablet return, laboratory tests)	8 and 9 (recruitment)<br>11
	11d	Relevant concomitant care and interventions that are permitted or prohibited during the trial	11
Outcomes	12	Primary, secondary, and other outcomes, including the specific measurement variable (eg, systolic blood pressure), analysis metric (eg, change from baseline, final value, time to event), method of aggregation (eg, median, proportion), and time point for each outcome. Explanation of the clinical relevance of chosen efficacy and harm outcomes is strongly recommended	11-16

For peer review only - http://bmjopen.bmj.com/site/about/guidelines.xhtml

Participant timeline	13	Time schedule of enrolment, interventions (including any run-ins and washouts), assessments, and visits for participants. A schematic diagram is highly recommended (see Figure)	Figure 2. Study Schedule diagram
Sample size	14	Estimated number of participants needed to achieve study objectives and how it was determined, including clinical and statistical assumptions supporting any sample size calculations	11<br>Primary outcome is recruitment rate for this feasibility trial so no formal sample size.
Recruitment	15	Strategies for achieving adequate participant enrolment to reach target sample size	8 and 9 (recruitment strategy)
**Methods: Assignment of interventions (for controlled trials)**			
Allocation:			
Sequence generation	16a	Method of generating the allocation sequence (eg, computer-generated random numbers), and list of any factors for stratification. To reduce predictability of a random sequence, details of any planned restriction (eg, blocking) should be provided in a separate document that is unavailable to those who enrol participants or assign interventions	6 (randomisation of GP practices)
Allocation concealment mechanism	16b	Mechanism of implementing the allocation sequence (eg, central telephone; sequentially numbered, opaque, sealed envelopes), describing any steps to conceal the sequence until interventions are assigned	8<br>The GP practices will be randomised 1:1 and stratified by CCG using Statarand (25), a Stata randomisation module (Boston College Department of Economics).
Implementation	16c	Who will generate the allocation sequence, who will enrol participants, and who will assign participants to interventions	7 and 8<br>Cluster randomised at level of GP practice.
Blinding (masking)	17a	Who will be blinded after assignment to interventions (eg, trial participants, care providers, outcome assessors, data analysts), and how	N/A
	17b	If blinded, circumstances under which unblinding is permissible, and procedure for revealing a participant's allocated intervention during the trial	N/A

For peer review only - http://bmjopen.bmj.com/site/about/guidelines.xhtml

**Methods: Data collection, management, and analysis**			
Data collection methods	18a	Plans for assessment and collection of outcome, baseline, and other trial data, including any related processes to promote data quality (eg, duplicate measurements, training of assessors) and a description of study instruments (eg, questionnaires, laboratory tests) along with their reliability and validity, if known. Reference to where data collection forms can be found, if not in the protocol	12
	18b	Plans to promote participant retention and complete follow-up, including list of any outcome data to be collected for participants who discontinue or deviate from intervention protocols	5<br>One of the feasibility aims is to assess retention rate.
Data management	19	Plans for data entry, coding, security, and storage, including any related processes to promote data quality (eg, double data entry; range checks for data values). Reference to where details of data management procedures can be found, if not in the protocol	17<br>Also detailed in the PIS (uploaded as supplementary material)
Statistical methods	20a	Statistical methods for analysing primary and secondary outcomes. Reference to where other details of the statistical analysis plan can be found, if not in the protocol	17 and 18
	20b	Methods for any additional analyses (eg, subgroup and adjusted analyses)	
	20c	Definition of analysis population relating to protocol non-adherence (eg, as randomised analysis), and any statistical methods to handle missing data (eg, multiple imputation)	
**Methods: Monitoring**			
Data monitoring	21a	Composition of data monitoring committee (DMC); summary of its role and reporting structure; statement of whether it is independent from the sponsor and competing interests; and reference to where further details about its charter can be found, if not in the protocol. Alternatively, an explanation of why a DMC is not needed	Data will be monitored, checked and cleaned by the study team for this feasibility study.
	21b	Description of any interim analyses and stopping guidelines, including who will have access to these interim results and make the final decision to terminate the trial	
Harms	22	Plans for collecting, assessing, reporting, and managing solicited and spontaneously reported adverse events and other unintended effects of trial interventions or trial conduct	9 and 10

For peer review only - http://bmjopen.bmj.com/site/about/guidelines.xhtml

Auditing	23	Frequency and procedures for auditing trial conduct, if any, and whether the process will be independent from investigators and the sponsor	The University of Leicester, as Sponsor, operates a risk based monitoring and audit programme, to which this study will be subject.
**Ethics and dissemination**			
Research ethics approval	24	Plans for seeking research ethics committee/institutional review board (REC/IRB) approval	3<br>The Research Ethics Committee Nottingham 1 has provided ethical approval for this research study (REC Reference: 19/EM/0201).
Protocol amendments	25	Plans for communicating important protocol modifications (eg, changes to eligibility criteria, outcomes, analyses) to relevant parties (eg, investigators, REC/IRBs, trial participants, trial registries, journals, regulators)	18
Consent or assent	26a	Who will obtain informed consent or assent from potential trial participants or authorised surrogates, and how (see Item 32)	10<br>Informed consent will be obtained from members of the study team.
	26b	Additional consent provisions for collection and use of participant data and biological specimens in ancillary studies, if applicable	N/A
Confidentiality	27	How personal information about potential and enrolled participants will be collected, shared, and maintained in order to protect confidentiality before, during, and after the trial	12 and 17
Declaration of interests	28	Financial and other competing interests for principal investigators for the overall trial and each study site	20<br>Authors declare no competing interests.
Access to data	29	Statement of who will have access to the final trial dataset, and disclosure of contractual agreements that limit such access for investigators	Detailed in the PIS uploaded as supplementary material.

For peer review only

For peer review only - http://bmjopen.bmj.com/site/about/guidelines.xhtml

Ancillary and post-trial care	30	Provisions, if any, for ancillary and post-trial care, and for compensation to those who suffer harm from trial participation	9<br>Participation is considered to be very low risk.
Dissemination policy	31a	Plans for investigators and sponsor to communicate trial results to participants, healthcare professionals, the public, and other relevant groups (eg, via publication, reporting in results databases, or other data sharing arrangements), including any publication restrictions	19-20
	31b	Authorship eligibility guidelines and any intended use of professional writers	
	31c	Plans, if any, for granting public access to the full protocol, participant-level dataset, and statistical code	
**Appendices**			
Informed consent materials	32	Model consent form and other related documentation given to participants and authorised surrogates	Uploaded Appendix A and B
Biological specimens	33	Plans for collection, laboratory evaluation, and storage of biological specimens for genetic or molecular analysis in the current trial and for future use in ancillary studies, if applicable	N/A

*It is strongly recommended that this checklist be read in conjunction with the SPIRIT 2013 Explanation & Elaboration for important clarification on the items. Amendments to the protocol should be tracked and dated. The SPIRIT checklist is copyrighted by the SPIRIT Group under the Creative Commons "Attribution-NonCommercial-NoDerivs 3.0 Unported" license.

For peer review only

For peer review only - http://bmjopen.bmj.com/site/about/guidelines.xhtml
